# Applications of the Multiattribute Decision-Making for the Development of the Tourism Industry Using Complex Intuitionistic Fuzzy Hamy Mean Operators

**DOI:** 10.1155/2022/8562390

**Published:** 2022-10-10

**Authors:** Abrar Hussain, Kifayat Ullah, Jihad Ahmad, Hanen Karamti, Dragan Pamucar, Haolun Wang

**Affiliations:** ^1^Department of Mathematics, Riphah International University (Lahore Campus), Lahore 54000, Pakistan; ^2^Department of Mathematics, Aden University, Aden, P. O. Box 6014, Yemen; ^3^Department of Computer Sciences, College of Computer and Information Sciences, Princess Nourah bint Abdulrahman University, P.O.Box 84428, Riyadh 11671, Saudi Arabia; ^4^Department of Operations Research and Statistics, Faculty of Organizational Sciences, University of Belgrade, Belgrade, Serbia; ^5^School of Economics & Management, Nanchang Hangkong Universtiy, Nanchang 330036, China

## Abstract

In the aggregation of uncertain information, it is very important to consider the interrelationship of the input information. Hamy mean (HM) is one of the fine tools to deal with such scenarios. This paper aims to extend the idea of the HM operator and dual HM (DHM) operator in the framework of complex intuitionistic fuzzy sets (CIFSs). The main benefit of using the frame of complex intuitionistic fuzzy CIF information is that it handles two possibilities of the truth degree (TD) and falsity degree (FD) of the uncertain information. We proposed four types of HM operators: CIF Hamy mean (CIFHM), CIF weighted Hamy mean (CIFWHM), CIF dual Hamy mean (CIFDHM), and CIF weighted dual Hamy mean (CIFWDHM) operators. The validity of the proposed HM operators is numerically established. The proposed HM operators are utilized to assess a multiattribute decision-making (MADM) problem where the case study of tourism destination places is discussed. For this purpose, a MADM algorithm involving the proposed HM operators is proposed and applied to the numerical example. The effectiveness and flexibility of the proposed method are also discussed, and the sensitivity of the involved parameters is studied. The conclusive remarks, after a comparative study, show that the results obtained in the frame of CIFSs improve the accuracy of the results by using the proposed HM operators.

## 1. Introduction

MADM is an essential process of decision-making (DM) science whose objective is to see the best options from the arrangement of likely ones. In DM, a singular requirement is to evaluate the given choices by different classes, such as single, range, and so for appraisal purposes. Nevertheless, in various fanciful conditions, it is, for the most part, pursuing for the person to convey their choices as a new number. For this, the frame of the fuzzy set (FS) [1] was developed. FS is a significant device for managing problematic and complex data in everyday normal life issues, and various analysts have utilized it in various fields. Notwithstanding, now and again, the hypothesis of FS is not equipped for managing such a sort of worry; for instance, if someone gives specific knowledge of the information, including the level of TD and lie, then, at that point, the hypothesis of FS becomes unable to be applied. To manage such issues, Atanassov [2] generalized the idea of FS to an intuitionistic fuzzy set (IFS) by utilizing the sum of TD and FD on the interval [0,1].

Sometimes, the information has more than one aspect. For example, when we want to purchase a laptop, there may be many things one can keep in mind such as its RAM, ROM, its generation, its price, and so on. To express single information having two aspects, Ramot et al. [3] gave the theory of complex FS (CFS) where the TD describes two aspects of uncertain phenomena by using a complex number having a magnitude less than or equal to 1. A complex fuzzy number (CFN) has the form *r*_*μ*_*e*^2*πiθμ*^ where *r*_*μ*_, *θμ* ∈ [0,1] and such kind of framework describes two different aspects of an uncertain phenomena. Moreover, the idea of CFS recently got a large number of attractions, and some useful work may be found in [4–7]. Consequently, Alkouri and Salleh [8] enhanced the concept of CFS by introducing the notion of complex IFS (CIFS) by adding the FD into the evaluation. A CIFN having two further aspects of the TD and FD of a phenomenon, known by amplitude and phase terms related with concurring values, represents the strength of suitable information, and the phase terms require additional information, which is associated with periodicity. The notion of CIFS has been applied to several real-life problems based on aggregation operators (AOs) and information measures. Gulzar et al. [9] utilized the notion of CIFSs in group theory. Rajareega et al. [10] analyzed and proposed some distance measures in the frame of CIF soft lattice ordered sets. Khalaf et al. [11] worked on similarity measures of temporal CIFSs and analyzed the structure of temporal CIFSs. Akram et al. [12] worked on CIF Hamacher AOs and used a DM model to solve the problem of electricity generation. Garg et al. [13] defined new geometric AOs by using t-norm rules in the environment of CIFS. Al-Hasban et al. [14] discovered the idea of a CIF normal subgroup and investigate a numerical example by using a DM method.

Aggregation of information is a significant tool that has some widespread applications especially when it comes to MADM problems. There are several AOs that are used for MADM problems including weighted averaging (WA) and weighted geometric (WG) AOs [15], Einstein WA operator and Einstein WG AOs [16, 17], Dombi WA and Dombi WG AOs [18], Pythagorean fuzzy (PyF) Aczel Alsina (PyFAA) AOs [19], interval-valued PyFAA AOs [20], Einstein geometric AOs [21], AOs of picture FSs [22], and Hamacher AOs [23, 24]. In a decision-making (DM) problem, the mutual relationship of the information has created a certain impact on results. The above-discussed AOs do not discuss the relationship of the information being aggregated and are hence unable to provide reliable information.

To deal with problems in a reliable way, the concept of the Hamy mean (HM) operator is noticeable. First of all, Hara et al. [25] introduced the concepts of HM operators to investigate the correlation of any numbers among them by using the various parameters. Qin [26] extended the concept of the HM operator in the framework of interval type 2 FS for DM. After that, Wu et al. [27] worked on the HM operator in the environment of interval-valued IFS (IVIFS) and develop the HM and weighted HM operators for MADM purposes. Wu et al. [28] investigated the service quality in tourism by using Dombi t-norm-based HM operators through a MADM approach under the environment of IVIFSs. Wang et al. [29] utilized the idea of HM operator under q-rung orthopair FS (q-ROFS) for the selection of enterprise resource planning. Liu et al. [30] analyzed the neutrosophic power HM operator based on vague information that exists in real-life problems. Liu and Wang [31] of IFSs and proposed some interactive HM operators for IFSs. Sinani et al. [32] introduced some Dombi-based HM operators in rough set theory and utilized them in the evaluation of logistics providers. Liu and Liu [33] studied the linguistic IFSs and developed some HM operators for MADM problems. Liu and You [34] investigated the linguistic HM operators in neutrosophic settings for MADM. Liang [35] gave a series of new AOs using the HM theory based on IFSs. The Concepts of Hesitant fuzzy linguistic power HM AOs were discovered by Liu et al. [36]. AOs of Dual Hesitant PyF HM operators were proposed by Wei et al. [37]. Wei et al. [38] proposed the notion of power HM operators for 2-tuple linguistic picture FSs and studied their applications in MADM, and Li et al. [35] introduced the idea of intuitionistic fuzzy (IF) Dombi HM operator and solved the Multi attributes group DM problem to carry out the selection of most suitable car for a transportation company. Some other work on HM operators can be found in [40, 41].

CIFSs have information in the form of TD and FD. Furthermore, TD has two aspects: amplitude terms and phase terms. Similarly, FD also has two aspects: amplitude terms and phase terms. CIFSs are the extension of FSs, CFSs, and IFSs. Both aspects of amplitude and phase terms of the parameters are distinguished between IFSs and CIFSs. Keeping in mind the significance of HM operators and the diverse nature of CIFSs, our target is to introduce HM operators into the layout of CIFS. The main advantage of doing such work is to consider the relationship of the decision preferences and to handle complex situations where the uncertain information is described by using the CIFSs where the TD and FD of fuzzy information have further two aspects. The main features of this article are as follows:

1. Utilizing the concepts of HM operators under the environment of CIFSs, we established AOs of CIFHM operators
2. We study properties of CIFHM operators such as idempotency, monotonicity, and boundedness
3. To evaluate the techniques of the MADM process, we established an application with the help of numerical examples based on the tourism industry in which the selection of the best tourism destination is carried out
4. We show the compatibility of the proposed HM operators by comparing the results with other existing AOs operators.

This paper is organized as follows: In Section 1, we evoke the previous background of the proposed work and discuss the research gap. In Section 2, we recall the basic definitions of IFS, CIFS, and some of their properties. In Section 3, we recall the idea of HM operators and some previously existing HM operators of IFSs and PyFSs. We also discussed the limitations of the previous HM operators in this section. In Section 4, we use the idea of HM operator in the environment of CIFS and develop the concepts of CIFHM and CIFWHM operator. In Section 5, we generalize the idea of a dual HM (DHM) operator in the environment of CIFS and introduce CIFDHM and CIFWDHM operators with some necessary conditions. In Section 6, we apply the HM operator of CIFS in a MADM problem where the problem of the selection of the best tourism destination is thoroughly discussed and investigated. We also study the impact of associated parameters on the ranking results and check the sensitivity of the proposed HM operators. In Section 7, we compare the results obtained using the proposed HM operator of CIFS and some other AOs of the CIFSs to study the reliability of the proposed operator. We conclude the manuscript with some significant remarks and future plans in Section 8.

## 2. Preliminaries

In this section, we recall some basic concepts of FS, IFS, CFS, and CIFS along with other notions. A nonempty set *H* is the universal set, and *μ*(*x*) and *ν*(*x*) represent the TD and FD, respectively, in this whole article.

**Definition 1 (see [2]).**

An IFS is of the form of *Ç*={(*x*, *μ*_*Ç*_(*x*), *ν*_*Ç*_(*x*))*|x* ∈ *y*}, where *μ*_*Ç*_(*x*) : *Y*⟶[0,1] and *ν*_*Ç*_(*x*) : *Y*⟶[0,1] provided that 0 ≤ *μ*_*Ç*_(*x*)+*ν*_*Ç*_(*x*) ≤ 1 and hesitancy degree represent *v*_c_(*x*)=1 − (*μ*_*Ç*_(*x*)+*ν*_*Ç*_(*x*)), (*x*) ∈ [0,1]. Furthermore, *Ç*=(*r*_*μ*_*Ç*__, *s*_*ν*_*Ç*__(*x*)) denotes an intuitionistic fuzzy number CIFN.

**Definition 2 (see [42]).**

A CIFS is of the form of *Ç* = {(*x*, *μ*_*Ç*_(*x*), *ν*_*Ç*_(*x*))*|x* ∈ *y*} such that *μ*_*Ç*_(*x*) = *r*_*μ*_*Ç*__(*x*)*e*^2*πiθ*_*μ*_*Ç*__(*x*)^ and *ν*_*Ç*_(*x*) = *s*_*ν*_*Ç*__(*x*)*e*^2*πiΦ*_*ν*_*Ç*__(*x*)^ where *r*_*μ*_*Ç*__(*x*), *Φs*_*ν*_*Ç*__(*x*) denote the amplitude terms and *θ*_*μ*_*Ç*__(*x*)*Φ*,_*ν*_*Ç*__(*x*) denote the phase terms of *μ*_*Ç*_(*x*) and *ν*_*Ç*_(*x*), respectively, from [0,1] provided that 0 ≤ *r*_*μ*_*Ç*__(*x*) + *s*_*ν*_*Ç*__(*x*) ≤ 1 and 0 ≤ *θ*_*μ*_*Ç*__(*x*) + *Φs*_*ν*_*Ç*__(*x*) ≤ 1 and hesitancy degree represent ℏ(*x*) = 1 − (*r*_*μ*_*Ç*__(*x*)*e*^2*πiθ*_*μ*_*Ç*__(*x*)^ + *s*_*ν*_*Ç*__(*x*)*e*^2*πi*_*ν*_*Ç*__(*x*)^), ℏ(*x*) ∈ [0,1]. Furthermore, *Ç* = (*r*_*μ*_*Ç*__(*x*)*e*^2*πiθ*_*μ*_*Ç*__(*x*)^, *s*_*ν*_*Ç*__(*x*)*e*^2*πiΦ*_*ν*_*Ç*__(*x*)^) represents a complex intuitionistic fuzzy number CIFN.

**Definition 3 (see [42]).**

Let*Ç* = (*r*_*μ*_*Ç*__(*x*)*e*^2*πiθ*_*μ*_*Ç*__(*x*)^, *s*_*ν*_*Ç*__(*x*)*e*^2*πi*_*ν*_*Ç*__(*x*)^) be a CIFN. The score function *S* is as follows:

(1) $$\begin{array}{c} S\left(Ç\right)=\frac{1}{2}\left(\left(r_{\mu_{Ç}}\left(x\right)-s_{\nu_{Ç}}\left(x\right)\right)+\left(\theta_{\mu_{Ç}}\left(x\right){-}_{{\Phi \nu }_{Ç}}\left(x\right)\right)\right),S\left(Ç\right)\in \left[-1,1\right]. \end{array}$$

**Definition 4 (see [42]).**

Let*Ç* = (*r*_*μ*_*Ç*__(*x*)*e*^2*πiθ*_*μ*_*Ç*__(*x*)^, *r*_*ν*_*Ç*__(*x*)*e*^2*πiΦ*_*ν*_*Ç*__(*x*)^) be a CIFN. The accuracy function ′*H* is as follows:

(2) $$\begin{array}{c} H\left(Ç\right)=\frac{1}{2}\left(\left(r_{\mu_{Ç}}\left(x\right)+r_{\nu_{Ç}}\left(x\right)\right)+\left(\theta_{\mu_{Ç}}\left(x\right)+{\Delta \Phi }_{\nu_{Ç}}\left(x\right)\right)\right),H\left(Ç\right)\in \left[0,1\right]. \end{array}$$

We gave Example 1 to support Definitions 3 and 4.

**Example 1 .**

Let *Ç*_1_=(0.3*e*^*i*2*π*(0.45)^, 0.5*e*^*i*2Π(0.25)^) and *Ç*_2_=(0.6*e*^*i*2Π(0.70)^, 0.2*e*^*i*2Π(.25)^) be two CIFNs. By using Definitions 3 and 4, we get the following:

 
š(*Ç*_1_)=1/2((0.3 − 0.5)+(0.45 − 0.25))=1/2(−0.20+0.20)=0 ∈ [−1,1]
 
š(*Ç*_2_)=1/2((0.6 − 0.2)+(0.70 − 0.25))=1/2(0.40+0.45)=0.48 ∈ [−1,1]
 
*H*(*Ç*_1_)=1/2((0.3+0.5)+(0.45+0.25))=1/2(0.80+0.70)=0.75 ∈ [0,1]
 
*H*(*Ç*_2_)=1/2((0.6+0.2)+(0.70+0.25))=1/2(0.8+0.95)=0.88 ∈ [0,1]

**Remark 1 .**

Let *Ç*_1_ = (*r*_*μ*_1__(*x*)*e*^2*πiθ*_*μ*_1__(*x*)^, *r*_*ν*_1__(*x*)*e*^2*πiΦ*_*ν*_1__(*x*)^) and *Ç*_2_ = (*r*_*μ*_2__(*x*)*e*^2*πiθ*_*μ*_2__(*x*)^, *r*_*ν*_2__(*x*)*e*^2*πiΦ*_*ν*_2__(*x*)^) be two CIFNs. Then

1. If *S*(*Ç*_1_) < *S*(*Ç*_2_), then *Ç*_1_ < *Ç*_2_
2. If *S*(*Ç*_1_) > *S*(*Ç*_2_), then *Ç*_1_ > *Ç*_2_
3. If *S*(*Ç*_1_) = *S*(*Ç*_2_), then:
4. *H*(*Ç*_1_) > *H*(*Ç*_2_), then*Ç*_1_ > *Ç*_2_
5. *H*(*Ç*_1_) < *H*(*Ç*_2_), then*Ç*_1_ < *Ç*_2_
6. *H*(*Ç*_1_) = *H*(*Ç*_2_), then*Ç*_1_ ≈ *Ç*_2._

**Definition 5 (see [43]).**

Let*Ç*_1_ = (*r*_*μ*_1__(*x*)*e*^2*πiθ*_*μ*_1__(*x*)^, *r*_*ν*_1__(*x*)*e*^2*πiΦ*_*ν*_1__(*x*)^) and *Ç*_2_ = (*r*_*μ*_2__(*x*)*e*^2*πiθ*_*μ*_2__(*x*)^, *r*_*ν*_2__(*x*)*e*^2*πiΦ*_*ν*_2__(*x*)^) be two CIFNs. Then some fundamental operations are defined as follows:

1. *Ç*_1_⊆*Ç*_2_⟺*r*_*μ*_1__(*x*) ≤ *r*_*μ*_2__(*x*),  *θ*_*μ*_1__(*x*) ≤ *θ*_*μ*_2__(*x*) and *r*_*ν*_1__(*x*) ≥ *r*_*ν*_2__(*x*),  Φ_*ν*_1__(*x*) ≥ Φ_*ν*_2__(*x*)
2. *Ç*_1_ = *Ç*_2_⟺*r*_*μ*_1__(*x*) = *r*_*μ*_2__(*x*),  *θ*_*μ*_1__(*x*) = *θ*_*μ*_2__(*x*)*r*_*Ç*_1__(*x*) = *r*_*Ç*_2__(*x*), *θ*_*Ç*_1__(*x*) = Δ_*Ç*_2__(*x*) and *r*_*ν*_1__(*x*) = *r*_*ν*_2__(*x*),  *Φ*_*ν*_1__(*x*) = *Φ*_*ν*_2__(*x*)
3. ${Ç}_{1}\cup {Ç}_{2}=\left\{\left(\begin{array}{c} \left(r_{\mu_{1}}\left(x\right)\vee r_{\mu_{2}}\left(x\right)\right)e^{2\pi i\left(\theta_{\mu_{1}}\left(x\right)\vee \theta_{\mu_{2}}\left(x\right)\right)}\\ \left(r_{\nu_{1}}\left(x\right)\wedge r_{\nu_{2}}\left(x\right)\right)e^{2\pi i\left(\Phi_{\nu_{1}}\left(x\right)\wedge \Phi_{\nu_{2}}\left(x\right)\right)} \end{array}\right)\right\}$
4. ${Ç}_{1}\cap {Ç}_{2}=\left\{\left(\begin{array}{c} \left(r_{\mu_{1}}\left(x\right)\wedge r_{\mu_{2}}\left(x\right)\right)e^{2\pi i\left(\theta_{\mu_{1}}\left(x\right)\wedge \theta_{\mu_{2}}\left(x\right)\right)},\\ \left(r_{\nu_{1}}\left(x\right)\vee r_{\nu_{2}}\left(x\right)\right)e^{2\pi i\left(\Phi_{\nu_{1}}\left(x\right)\vee \Phi_{\nu_{2}}\left(x\right)\right)} \end{array}\right)\right\}$
5. *Ç*_1_′ = {(*r*_*ν*_1__(*x*)*e*^*iΦ*_*ν*_1__(*x*)^, *r*_*μ*_1__(*x*)*e*^*iθ*_*μ*_1__(*x*)^)}

**Definition 6 (see [42]).**

Let*Ç*_1_ = (*r*_*μ*_1__(*x*)*e*^2*πiθ*_*μ*_1__(*x*)^, *r*_*ν*_1__(*x*)*e*^2*πiΦ*_*ν*_1__(*x*)^) and *Ç*_2_ = (*r*_*μ*_2__(*x*)*e*^2*πiθ*_*μ*_2__(*x*)^, *r*_*ν*_2__(*x*)*e*^2*πiΦ*_*ν*_2__(*x*)^) be two CIFNs and *λ* > 0 be a real number. Then

1. ${Ç}_{1}⊕{Ç}_{2}=\left(\begin{array}{c} \left(r_{\mu_{1}}\left(x\right)+r_{\mu_{2}}\left(x\right)-r_{\mu_{1}}\left(x\right)\cdot r_{\mu_{2}}\left(x\right)\right)\cdot e^{2\pi i\left(\theta_{\mu_{1}}\left(x\right)+\theta_{\mu_{2}}\left(x\right)-\theta_{\mu_{1}}\left(x\right)\cdot \theta_{\mu_{2}}\left(x\right)\right)},\\ \left(r_{\nu_{1}}\left(x\right)\cdot r_{\nu_{2}}\left(x\right)\right)e^{2\pi i\left(\Phi_{\nu_{1}}\left(x\right)\cdot \Phi_{\nu_{2}}\left(x\right)\right)} \end{array}\right)$
2. ${Ç}_{1}⊕{Ç}_{2}=\left\{\begin{array}{c} \left(r_{\mu_{1}}\left(x\right)\cdot r_{\mu_{2}}\left(x\right)\right)e^{2\pi i\left(\theta_{\mu_{1}}\left(x\right)\cdot \theta_{\mu_{2}}\left(x\right)\right)},\\ \left(r_{\nu_{1}}\left(x\right)+r_{\nu_{2}}\left(x\right)-r_{\nu_{1}}\left(x\right)\cdot r_{\nu_{2}}\left(x\right)\right)\cdot e^{2\pi i\left(\Phi_{\nu_{1}}\left(x\right)+\Phi_{\nu_{2}}\left(x\right)-\Phi_{\nu_{1}}\left(x\right)\cdot \Phi_{\nu_{2}}\left(x\right)\right)} \end{array}\right\}$
3. *λÇ*_1_ = (1 − (1 − *r*_*μ*_1__(*x*))^*λ*^*e*^2*πi*(1 − (1 − *θ*_*μ*_1__(*x*))^*λ*^)^, (*r*_*ν*_1__^*λ*^(*x*) · *e*^2*πi*(*Φ*_*ν*_1__(*x*))^*λ*^^))
4. *Ç*_1_^*λ*^ = ((*r*_*μ*_1__^*λ*^(*x*)*e*^2*πi*(*θ*_*μ*_1__(*x*))^*λ*^^), 1 − (1 − *r*_*ν*_1__(*x*))^*λ*^*e*^2*πi*(1 − (1 − *Φ*_*ν*_1__(*x*))^*λ*^)^)

## 3. Previous Study

In this section, we recall the basic definition of HM operator. We also discuss the HM operators that are previously defined. Furthermore, we point out towards the limitations of such existing HM operators that lead us to propose some new HM operators.

Consider the HM operators defined for real numbers.

**Definition 7 (see [25]).**

The HM operator is as follows:

(3) $$\begin{array}{c} {\text{HM}}^{\left(x\right)}\left({Ç}_{1},{Ç}_{2},\ldots ,{Ç}_{n}\right)=\frac{\sum_{1\leq i_{1}<\cdots <i_{x}\leq n}{\left(\prod_{i=1}^{x}{Ç}_{i_{j}}\right)}^{1/x}}{C_{n}^{x}}, \end{array}$$

where *x* is such that 1 ≤ *x* ≤ *n* and *C*_*k*_^*x*^ represent the binomial coefficient, that is, *C*_*n*_^*x*^ = *x*!/*n*!(*x* − *n*)!

The HM operator is likely to satisfy the following properties:

1. HM^(*x*)^(*Ç*_1_, *Ç*_2_,…, *Ç*_*k*_)=*Ç* if *Ç*_*i*_=*Ç*, (*i*=1,2,3,…, *k*)
2. HM^(*x*)^(*Ç*_1_, *Ç*_2_,…, *Ç*_*k*_) ≤ HM^(*x*)^(*ω*_1_, *ω*_2_,…, *ω*_*k*_) if *Ç*_*i*_ ≤ *ω*_*i*_, (*i*=1,2,3,…, *k*)
3. min(*Ç*_*i*_) ≤ *HM*^(*x*)^(*Ç*_1_, *Ç*_2_,…, *Ç*_*k*_) ≤ max*Ç*_*i*_
4. For arithmetic mean operator HM^(*x*)^(*Ç*_1_, *Ç*_2_,…, *Ç*_*k*_)=(1/*k*)∑_*i*=1_^*k*^*Ç*_*i*_
5. For geometric mean operator HM^(*x*)^(*Ç*_1_, *Ç*_2_,…, *Ç*_*k*_)=(∏_*i*=1_^*k*^*Ç*_*i*_)^1/*x*^

We recall the definition of the DHM operator for real numbers.

**Definition 8 (see [44]).**

The DHM operator is defined as follows:

(4) $$\begin{array}{c} {\text{DHM}}^{\left(x\right)}\left({Ç}_{1},{Ç}_{2},\ldots ,{Ç}_{n}\right)={\left(\underset{1\leq i_{1}<\cdots <i_{x}\leq n}{\prod }\left(\frac{\sum_{j=1}^{x}{Ç}_{j}}{x}\right)\right)}^{1/C_{n}^{x}}. \end{array}$$

**Definition 9 (see [31]).**

Let *I*_*j*_ = (*r*_*μ*_*j*__(*x*), *s*_*j*_(*x*)), *j* = 1,2,…, *k* be the collection of IFNs. Then intuitionistic fuzzy Hamy mean (IFHM) operator is defined as follows:

(5) $$\begin{array}{c} {\text{IFHM}}^{\left(x\right)}\left({Ç}_{1},{Ç}_{2},\ldots ,{Ç}_{n}\right)=\frac{{⊕}_{1\leq i_{1}<\cdots <i_{x}\leq n}{\left({⊕}_{i=1}^{x}I_{i_{j}}\right)}^{1/x}}{C_{n}^{x}} \end{array}$$

(6) $$\begin{array}{c} =\left(1-{\left(\underset{1\leq i_{1}<\cdots <i_{x}\leq n}{\prod }\left(1-{\left(\overset{x}{\underset{j=1}{\prod }}r_{\mu_{j}}\left(x\right)\right)}^{1/x}\right)\right)}^{1/C_{n}^{x}}{\left(\underset{1\leq i_{1}<\cdots <i_{x}\leq n}{\prod }\left(1-{\left(\overset{x}{\underset{j=1}{\prod }}\left(1-s_{\nu_{j}}\left(x\right)\right)\right)}^{1/x}\right)\right)}^{1/C_{n}^{x}}\right). \end{array}$$

**Definition 10 (see [27]).**

Let*I*_*j*_ = ([*r*_*j*_(*x*), *s*_*j*_(*x*)], [*t*_*j*_(*x*), *u*_*j*_(*x*)]), *j* = 1,2,…, *k* be the collection of interval-valued IFNs (IVIFNs). Then interval-valued intuitionistic fuzzy Hamy mean operator is defined as follows:

(7) $$\begin{array}{c} {\text{IVIFHM}}^{\left(x\right)}\left({Ç}_{1},{Ç}_{2},\ldots ,{Ç}_{n}\right)=\frac{{⊕}_{1\leq i_{1}<\cdots <i_{x}\leq n}{\left({⊕}_{i=1}^{x}I_{i_{j}}\right)}^{1/x}}{C_{n}^{x}},\\ {\text{IVIFHM}}^{\left(x\right)}\left({Ç}_{1},{Ç}_{2},\ldots ,{Ç}_{n}\right)=\left(\begin{array}{c} \left[1-{\left(\underset{1\leq i_{1}<\cdots <i_{x}\leq n}{\prod }\left(1-{\left(\overset{x}{\underset{j=1}{\prod }}r_{\mu_{j}}\left(x\right)\right)}^{1/x}\right)\right)}^{1/C_{n}^{x}},1-{\left(\underset{1\leq i_{1}<\cdots <i_{x}\leq n}{\prod }\left(1-{\left(\overset{x}{\underset{j=1}{\prod }}s_{\nu_{j}}\left(x\right)\right)}^{1/x}\right)\right)}^{1/C_{n}^{x}}\right],\;\\ \left[{\left(\underset{1\leq i_{1}<\cdots <i_{x}\leq n}{\prod }\left(1-{\left(\overset{x}{\underset{j=1}{\prod }}\left(1-t_{\mu_{j}}\left(x\right)\right)\right)}^{1/x}\right)\right)}^{1/C_{n}^{x}},{\left(\underset{1\leq i_{1}<\cdots <i_{x}\leq n}{\prod }\left(1-{\left(\overset{x}{\underset{j=1}{\prod }}\left(1-u_{\nu_{j}}\left(x\right)\right)\right)}^{1/x}\right)\right)}^{1/C_{n}^{x}}\right] \end{array}\right). \end{array}$$

All the above-discussed HM operators deal with two real values TD and FD. Consider a scenario with TD and FD having further two aspects, then the operators discussed above become unable to deal with such information. Therefore, we aim to propose the concept of HM operator in the framework of CIFS because such an operator can deal with two aspects of TD and FD at a time.

## 4. Complex Fuzzy Hamy Mean Operator

In this section, we introduced the idea of HM operators in the framework of CIFSs. We also proved that the CIFHM operator satisfied the basic properties of AO. We give example to support the proposed operator. First, consider the HM operators based on CIFNs as follows.

**Definition 11 .**

Let *Ç*_*j*_=(*r*_*μ*_*j*__(*x*)*e*^*iθ*_*μ*_*j*__(*x*)^, *s*_*v*_*j*__(*x*)*e*^*iΦ*_*ν*_*j*__(*x*)^), *j*=1,2,…, *k*, be the collection of CIFNs. Then, the CIFHM operator is defined as follows:

(8) $$\begin{array}{c} {\text{CIFHM}}^{\left(x\right)}\left({Ç}_{1},{Ç}_{2},\ldots ,{Ç}_{n}\right)=\frac{{⊕}_{1\leq i_{1}<\cdots <i_{x}\leq n}{\left({⊕}_{i=1}^{x}{Ç}_{j}\right)}^{1/x}}{C_{n}^{x}}. \end{array}$$

**Theorem 1 .**

Let *Ç*_*j*_=(*r*_*μ*_*j*__(*x*)*e*^2*πiθ*_*μ*_*j*__(*x*)^, *s*_*ν*_*j*__*e*^2*πiΦ*_*ν*_*j*__(*x*)^), *j*=1,2,…, *k* be the collection of CIFNs. Then the aggregated value of the CIFHM operator is also a CIFN such that

(9) $$\begin{array}{c} {\text{CIFHM}}^{\left(x\right)}\left({Ç}_{1},{Ç}_{2},\ldots ,{Ç}_{n}\right)=\left(\begin{array}{c} 1-{\left(\underset{1\leq i_{1}<\cdots <i_{x}\leq n}{\prod }\left(1-{\left(\overset{x}{\underset{j=1}{\prod }}r_{\mu_{j}}\left(x\right)\right)}^{1/x}\right)\right)}^{1/C_{n}^{x}}\cdot e^{2\pi i\;\left(1-{\left(\prod_{1\leq i_{1}<\cdots <i_{x}\leq n}\left(1-{\left(\prod_{j=1}^{x}\theta_{\mu_{j}}\left(x\right)\right)}^{1/x}\right)\right)}^{1/C_{n}^{x}}\right)}\\ {\left(\underset{1\leq i_{1}<\cdots <i_{x}\leq n}{\prod }\left(1-{\left(\overset{x}{\underset{j=1}{\prod }}\left(1-s_{\nu_{j}}\left(x\right)\right)\right)}^{1/x}\right)\right)}^{1/C_{n}^{x}}\cdot e^{2\pi i\;\left({\left(\prod_{1\leq i_{1}<\cdots <i_{x}\leq n}\left(1-{\left(\prod_{j=1}^{x}\left(1-\Phi_{\nu_{j}}\left(x\right)\right)\right)}^{1/x}\right)\right)}^{1/C_{n}^{x}}\right)} \end{array}\right). \end{array}$$

**Proof**

This theorem has two parts: first, we derive the formula given in equation (6) as follows:

(10) $$\begin{array}{c} \overset{x}{\underset{i=1}{⊕}}{Ç}_{j}=\left(\overset{x}{\underset{j=1}{\prod }}r_{\mu_{j}}\left(x\right)e^{2\pi i\left(\prod_{j=1}^{x}\theta_{\mu_{j}}\left(x\right)\right)},\left(1-\overset{x}{\underset{j=1}{\prod }}\left(1-s_{\nu_{j}}\left(x\right)\right)\right)\cdot e^{2\pi i\;\left(1-\prod_{j=1}^{x}\left(1-\Phi_{\nu_{j}}\left(x\right)\right)\right)}\right),\\ {\left(\overset{x}{\underset{i=1}{⊕}}{Ç}_{j}\right)}^{1/x}=\left(\begin{array}{c} \left({\left(\overset{x}{\underset{j=1}{\prod }}r_{\mu_{j}}\left(x\right)\right)}^{1/x}e^{2\pi i{\left(\prod_{j=1}^{x}\theta_{\mu j}\left(x\right)\right)}^{1/x}}\right)\\ \left(1-\overset{x}{\underset{j=1}{\prod }}{\left(1-s_{\nu_{j}}\left(x\right)\right)}^{1/x}e^{2\pi i{\left(1-\prod_{j=1}^{x}\left(1-\Phi_{\nu_{j}}\left(x\right)\right)\right)}^{1/x}}\right) \end{array}\right),\\ \begin{array}{c} ⊕\\ 1\leq i_{t}<\cdots <i_{t} \end{array}{\left(\overset{x}{\underset{i=1}{⊕}}{Ç}_{j}\right)}^{1/x}=\left(\begin{array}{c} \left(1-\underset{1\leq i_{1}<\cdots <i_{t}}{\prod }\left(1-{\left(\overset{x}{\underset{j=1}{\prod }}r_{\mu_{j}}\left(x\right)\right)}^{1/x}\right)e^{2\pi i\left(1-\prod_{1\leq i_{t}<\cdots <i_{t}}\left(1-{\left(\prod_{j=1}^{x}\theta_{\mu j}\left(x\right)\right)}^{1/x}\right)\right)}\right)\\ \left(\underset{1\leq i_{1}<\cdots <i_{t}}{\prod }\left(1-\overset{x}{\underset{j=1}{\prod }}{\left(s_{\nu_{j}}\left(x\right)\right)}^{1/x}\right)e^{2\pi i\left(\prod_{1\leq i_{t}<\cdots <i_{t}}\left(1-{\left(\prod_{j=1}^{x}\Phi_{\nu_{j}}\left(x\right)\right)}^{1/x}\right)\right)}\right) \end{array}\right),\\ {\text{CIFHM}}^{x}\left({Ç}_{1},{Ç}_{2},\ldots ,{Ç}_{n}\right)=\left(\begin{array}{c} 1-{\left(\underset{1\leq i_{1}<\cdots <i_{x}\leq n}{\prod }\left(1-{\left(\overset{x}{\underset{j=1}{\prod }}r_{\mu_{j}}\left(x\right)\right)}^{1/x}\right)\right)}^{1/C\left(x/n\right)}e^{2\pi i\left(1-{\left(\prod_{1\leq i_{1}<\cdots <i_{n}\leq n}\left(1-{\left(\prod_{j=1}^{x}\theta_{\mu j}\left(x\right)\right)}^{1/x}\right)\right)}^{1/C\left(x/n\right)}\right)}\\ {\left(\underset{1\leq i_{1}<\cdots <i_{x}\leq n}{\prod }\left(1-{\left(\overset{x}{\underset{j=1}{\prod }}\left(1-s_{\nu_{j}}\left(x\right)\right)\right)}^{1/x}\right)\right)}^{1/C\left(x/n\right)}e^{2\pi i{\left(\prod_{1\leq i_{1}<\cdots <i_{x}\leq n}\left(1-{\left(\prod_{j=1}^{x}\left(1{-}_{\Phi \nu_{j}}\left(x\right)\right)\right)}^{1/x}\right)\right)}^{1/C\left(x/n\right)}} \end{array}\right). \end{array}$$

Now, we prove that equation (6) represents a CIFN as follows:

(11) $$\begin{array}{c} r_{\mu }\left(x\right),s_{\nu }\left(x\right)\in \left[0,1\right],\theta_{\mu }\left(x\right),\Delta_{\nu }\left(x\right)\in \left[0,2\pi \right]. \end{array}$$

1. 0 ≤ *r*_*μ*_ (*x*)+*s*_*ν*_(*x*) ≤ 1 and 0 ≤ *θ*_*μ*_(*x*)+*Φ*_*ν*_(*x*) ≤ 1

(12) $$\begin{array}{c} r_{\mu }\;\left(x\right)=1-{\left(\underset{1\leq i_{1}<\cdots <i_{x}\leq n}{\prod }\left(1-{\left(\overset{x}{\underset{j=1}{\prod }}r_{\mu_{j}}\left(x\right)\right)}^{1/x}\right)\right)}^{1/C_{n}^{x}},\\ \theta_{\mu }\left(x\right)=\;\left(1-{\left(\underset{1\leq i_{1}<\cdots <i_{n}\leq n}{\prod }\left(1-{\left(\overset{x}{\underset{j=1}{\prod }}\theta_{\mu j}\left(x\right)\right)}^{1/x}\right)\right)}^{1/C_{n}^{x}}\right),\\ s_{\nu }\left(x\right)={\left(\underset{1\leq i_{1}<\cdots <i_{x}\leq n}{\prod }\left(1-{\left(\overset{x}{\underset{j=1}{\prod }}\left(1-s_{\nu_{j}}\left(x\right)\right)\right)}^{1/x}\right)\right)}^{1/C_{n}^{x}},\\ \Delta_{\nu }\left(x\right)={\left(\underset{1\leq i_{1}<\cdots <i_{x}\leq n}{\prod }\left(1-{\left(\overset{x}{\underset{j=1}{\prod }}\left(1-\Phi_{\nu_{j}}\left(x\right)\right)\right)}^{1/x}\right)\right)}^{1/C_{n}^{x}}. \end{array}$$

We know that 0 ≤ *r*_*μ*_(*x*) ≤ 1 and 0 ≤ *θ*_*μ*_(*x*) ≤ 1 We have

(13) $$\begin{array}{c} 0\leq r_{\mu }\left(x\right)e^{2\pi i\theta_{\mu }\left(x\right)}\leq 1,\\ 0\leq \overset{x}{\underset{j=1}{\prod }}r_{\mu_{j}}\left(x\right)e^{2\pi i\left(\prod_{j=1}^{x}\theta_{\mu_{j}}\left(x\right)\right)}\leq 1,\\ 0\leq \overset{x}{\underset{j=1}{\prod }}r_{\mu_{j}}\left(x\right)e^{2\pi i\left(\prod_{j=1}^{x}\theta_{\mu_{j}}\left(x\right)\right)}\leq 1,\\ 0\leq 1-{\left(\overset{x}{\underset{j=1}{\prod }}r_{\mu_{j}}\left(x\right)\right)}^{1/x}e^{2\pi i\left(1-{\left(\prod_{j=1}^{x}\theta_{\mu_{j}}\left(x\right)\right)}^{1/x}\right)}\leq 1,\\ 0\leq \underset{1\leq i_{1}<,\ldots ,<i_{x}\leq n}{\prod }\left(1-{\left(\overset{x}{\underset{j=1}{\prod }}r_{\mu_{j}}\left(x\right)\right)}^{1/x}\right).e^{2\pi i\left(\prod_{1\leq i_{1}<,\ldots ,<i_{x}\leq n}\left(1-{\left(\prod_{j=1}^{x}\theta_{\mu_{j}}\left(x\right)\right)}^{1/x}\right)\right)}\leq 1,\\ 0\leq {\left(1-\underset{1\leq i_{1}<,\ldots ,<i_{x}\leq n}{\prod }\left(1-{\left(\overset{x}{\underset{j=1}{\prod }}\left(1-r_{\mu_{j}}\left(x\right)\right)\right)}^{1/x}\right)\right)}^{1/C_{n}^{x}}.e^{2\pi i\left({\left(1-\prod_{1\leq i_{1}<,\ldots ,<i_{x}\leq n}\left(1-{\left(\prod_{j=1}^{x}\left(1-\theta_{\mu_{j}}\left(x\right)\right)\right)}^{1/x}\right)\right)}^{1/C_{n}^{x}}\right)}\leq 1. \end{array}$$

Similarly,

(14) $$\begin{array}{c} 0\leq s_{\nu }\left(x\right)e^{2\pi i\Phi_{\nu }\left(x\right)}\leq 1. \end{array}$$

Since 0 ≤ *r*_*μ*_(*x*)*e*^2*πiθ*_*μ*_(*x*)^ ≤ 1 and 0 ≤ *s*_*ν*_(*x*)*e*^2*πiΦ*_*ν*_(*x*)^ ≤ 1 so we have

(15) $$\begin{array}{c} 0\leq r_{\mu }\left(x\right)e^{2\pi i\theta_{\mu }\left(x\right)}+s_{\nu }\left(x\right)e^{2\pi i_{\Phi \nu }\left(x\right)}\leq 1. \end{array}$$

Now, we prove that the CIFHM operator satisfies the properties of the aggregation function in Theorems 2–4, respectively.

**Theorem 2 (Idempotency Property).**

Let*Ç*_*j*_=(*r*_*μ*_*j*__(*x*)*e*^*iθ*(*μ*/*j*)(*x*)^, *s*_*ν*_*j*__(*x*)*e*^*i*Δ_*ν*_*j*__(*x*)^), *j*=1,2,…, *k* be the collection of all identical values of CIFNs. Then

(16) $$\begin{array}{c} CIFHM^{x}\left({Ç}_{1},{Ç}_{2},\ldots ,{Ç}_{n}\right)=Ç. \end{array}$$

**Proof**

We know that *Ç*_*j*_=(*r*_*μ*_*j*__(*x*)*e*^*iθ*(*μ*/*j*)(*x*)^, *s*_*ν*_*j*__(*x*)*e*^*iΦ*_*ν*_*j*__(*x*)^)=(*r*(*x*)*e*^*iθ*(*x*)^, *s*(*x*)*e*^*iΦ*(*x*)^)=*Ç*, *j*=1,2,…, *k*. Then

(17) $$\begin{array}{c} {\text{CIFHM}}^{x}\left({Ç}_{1},{Ç}_{2},\ldots ,{Ç}_{n}\right)=\left(\begin{array}{c} 1-{\left(\underset{1\leq i_{1}<,\ldots ,<i_{x}\leq n}{\prod }\left(1-{\left(\overset{x}{\underset{j=1}{\prod }}r_{\mu_{j}}\left(x\right)\right)}^{1/x}\right)\;\right)}^{1/C_{n}^{x}}e^{2\pi i\left(1-{\left(\prod_{1\leq i_{1}<,\ldots ,<i_{n}\leq n}\left(1-{\left(\prod_{j=1}^{x}\theta_{\mu_{j}}\left(x\right)\right)}^{\frac{1}{x}}\right)\right)}^{1/C_{n}^{x}}\right)},\\ {\left(\underset{1\leq i_{1}<,\ldots ,<i_{x}\leq n}{\prod }\left(1-{\left(\overset{x}{\underset{j=1}{\prod }}\left(1-s_{\nu_{j}}\left(x\right)\right)\right)}^{1/x}\right)\right)}^{1/C_{n}^{x}}e^{2\pi i{\left(\prod_{1\leq i_{1}<,\ldots ,<i_{x}\leq n}\left(1-{\left(\prod_{j=1}^{x}\left(1-\Phi_{\nu_{j}}\left(x\right)\right)\right)}^{1/x}\right)\right)}^{1/C_{n}^{x}}} \end{array}\right)\\ =\left(1-{\left(1-{\left(r_{\mu }\left(x\right)\right)}^{1/x}\right)}^{1/C_{n}^{x}}e^{2\pi i\left(1-{\left(1-{\left(\theta_{\mu }\left(x\right)\right)}^{1/x}\right)}^{1/C_{n}^{x}}\right)},{\left(1-{\left(s_{\nu }\left(x\right)\right)}^{1/x}\right)}^{1/C_{n}^{x}}e^{2\pi i\left({\left(1-{\left(\Phi_{\nu }\left(x\right)\right)}^{1/x}\right)}^{1/C_{n}^{x}}\right)}\right)\\ =\left(r_{\mu }\left(x\right)e^{i\theta_{\mu }},s_{\nu }\left(x\right)e^{i\Delta_{\nu }}\right)=Ç. \end{array}$$

**Theorem 3 (Monotonicity Property).**

Let *Ç*_*j*_=(*r*_*μ*_*j*__(*x*)*e*^*iθμ*/*j*(*x*)^, *s*_*ν*_*j*__(*x*)*e*^*iΦ*_*ν*_*j*__(*x*)^), *j*=1,2,…, *k* and *D*_*j*_(*x*)=(*g*_*μ*_*j*__(*x*)*e*^*iαμ*/*j*(*x*)^, *h*_*ν*_ _*j*_(*x*)*e*^*iβν*/*j*(*x*)^), *j*=1,2,…, *k* be two sets of CIFNs. If *Ç*_*j*_(*x*) ≤ *D*_*j*_(*x*), that is, *r*_*μ*_*j*__(*x*) ≤ *g*_*μ*_*j*__(*x*), *θ*_*μj*_(*x*) ≤ *α*_*μ*_*j*__(*x*) and *s*_*νj*_(*x*) ≤ *h*_*νj*_(*x*), Φ_*νj*_(*x*) ≤ *β*_*νj*_(*x*) then

(18) $$\begin{array}{c} {\text{CIFHM}}^{x}\left({Ç}_{1},{Ç}_{2},\ldots ,{Ç}_{n}\right)\leq {\text{CIFHM}}^{x}\left(D_{1},D_{2},\ldots ,D_{n}\right). \end{array}$$

**Proof**

We know that *Ç*_*j*_ ≤ *D*_*j*_, that is, *r*_*μ*_*j*__(*x*) ≤ *g*_*μ*_*j*__(*x*), *θ*_*μj*_(*x*) ≤ *α*_*j*_(*x*) and *s*_*j*_(*x*) ≤ *h*_*j*_(*x*), *Φ*_*j*_(*x*) ≤ *β*_*j*_(*x*). Then

(19) $$\begin{array}{c} \overset{x}{\underset{j=1}{\prod }}r_{\mu_{j}}\left(x\right)e^{2\pi \text{i}\left(\prod_{j=1}^{x}\theta_{\mu_{j}}\left(x\right)\right)}\leq \overset{x}{\underset{j=1}{\prod }}g_{\mu_{j}}e^{2\pi \text{i}\left(\prod_{j=1}^{x}\alpha_{\mu_{j}}\left(x\right)\right)},\\ 1-{\left(\overset{x}{\underset{j=1}{\prod }}r_{\mu_{j}}\left(x\right)\right)}^{1/x}e^{2\pi i\left(1-{\left(\prod_{j=1}^{x}\theta_{\mu_{j}}\left(x\right)\right)}^{1/x}\right)}\geq 1-{\left(\overset{x}{\underset{j=1}{\prod }}g_{\mu_{j}}\left(x\right)\right)}^{1/x}e^{2\pi i\left(1-{\left(\prod_{j=1}^{x}\alpha_{\mu_{j}}\left(x\right)\right)}^{1/x}\right)}\\ \left(\underset{1\leq i_{1}<,\ldots ,<i_{n}\leq i_{n}}{\prod }{\left(1-{\left(\overset{x}{\underset{j=1}{\prod }}r_{\mu_{j}}\left(x\right)\right)}^{1/x}\right)}^{1/C_{n}^{x}}\right)e^{2\pi i\left(\prod_{1\leq i_{1}<,\ldots ,<i_{n}\leq i_{n}}{\left(1-{\left(\prod_{j=1}^{x}\theta_{\mu_{j}}\left(x\right)\right)}^{1/x}\right)}^{1/C_{n}^{x}}\right)}\geq \left(\underset{1\leq i_{1}<,\ldots ,<i_{n}\leq i_{n}}{\prod }{\left(1-{\left(\overset{x}{\underset{j=1}{\prod }}g_{\mu_{j}}\left(x\right)\right)}^{1/x}\right)}^{1/C_{n}^{x}}\right)e^{2\pi i\left({\prod_{1\leq i_{1}<,\ldots ,<i_{n}\leq i_{n}}\left(1-{\left(\prod_{j=1}^{x}\alpha_{\mu_{j}}\left(x\right)\right)}^{1/x}\right)}^{1/C_{n}^{x}}\right)}\\ 1-\left(\underset{1\leq i_{1}<,\ldots ,<i_{n}\leq i_{n}}{\prod }{\left(1-{\left(\overset{x}{\underset{j=1}{\prod }}r_{\mu_{j}}\left(x\right)\right)}^{1/x}\right)}^{1/C_{n}^{x}}\right)e^{2\pi i\left(1-\left(\prod_{1\leq i_{1}<,\ldots ,<i_{n}\leq i_{n}}{\left(1-{\left(\prod_{j=1}^{x}\theta_{\mu_{j}}\left(x\right)\right)}^{1/x}\right)}^{1/C_{n}^{x}}\right)\right)}\\ \leq 1-\left(\underset{1\leq i_{1}<,\ldots ,<i_{n}\leq i_{n}}{\prod }{\left(1-{\left(\overset{x}{\underset{j=1}{\prod }}g_{\mu_{j}}\left(x\right)\right)}^{1/x}\right)}^{1/C_{n}^{x}}\right)e^{2\pi i\left(1-\left(\prod_{1\leq i_{1}<,\ldots ,<i_{n}\leq i_{n}}{\left(1-{\left(\prod_{j=1}^{x}\alpha_{\mu_{j}}\left(x\right)\right)}^{1/x}\right)}^{1/C_{n}^{x}}\right)\right)\;}. \end{array}$$

According to equation (19), *r*_*μ*_*j*__(*x*)*e*^2*πiθ*_*μ*_(*x*)^ ≤ *ge*^2*πiα*_*μ*_(*x*)^. In a similar way, we can investigate the value of *s*_*ν*_*j*__(*x*)*e*^2*πiΦ*_*ν*_(*x*)^ ≥ *h*_*ν*_(*x*)*e*^2*πiβ*_*ν*_(*x*)^.

(1) If *r*_*μ*_*j*__(*x*)*e*^2*πiθ*_*μ*_(*x*)^ < *g*_*μ*_*j*__(*x*)*e*^2*πiα*_*μ*_(*x*)^ and *s*_*ν*_(*x*)*e*^2*πiΦ*_*ν*_(*x*)^ > *h*_*ν*_(*x*)*e*^2*πiβ*_*ν*_(*x*)^, then
  (20) $$\begin{array}{c} CIFHM^{x}\left({Ç}_{1},{Ç}_{2},\ldots ,{Ç}_{n}\right)<CIFHM^{x}\left(D_{1},D_{2},\ldots ,D_{n}\right), \end{array}$$
(2) If *r*_*μ*_*j*__(*x*)*e*^2*πiθ*_*μ*_(*x*)^=*g*_*μ*_*j*__(*x*)*e*^2*πiα*_*μ*_(*x*)^ and *s*_*ν*_(*x*)*e*^2*πiΦ*_*ν*_(*x*)^=*h*_*ν*_(*x*)*e*^2*πiβ*_*ν*_(*x*)^, then

(21) $$\begin{array}{c} CIFHM^{x}\left({Ç}_{1},{Ç}_{2},\ldots ,{Ç}_{n}\right)=CIFHM^{x}\left(D_{1},D_{2},\ldots ,D_{n}\right). \end{array}$$

**Theorem 4 (Boundedness Property).**

Let *Ç*_*j*_=(*r*_*μ*_*j*__(*x*)*e*^*iθ*_*μ*_ _*j*_(*x*)^, *s*_*ν*_ _*j*_(*x*)*e*^*iΦ*_*ν*_ _*j*_(*x*)^), *j*=1,2,…, *k* be the collection of CIFNs. If *Ç*_*j*_^−^=min(*Ç*_1_, *Ç*_2_, *Ç*_3_,…, *Ç*_*n*_) and *Ç*_*j*_^+^=max(*Ç*_1_, *Ç*_2_, *Ç*_3_,…, *Ç*_*n*_), then

(22) $$\begin{array}{c} {Ç}^{-}\leq CIFHM^{x}\left({Ç}_{1},{Ç}_{2},\ldots ,{Ç}_{n}\right)\leq {Ç}^{+}. \end{array}$$

**Proof**

From boundedness property:

(23) $$\begin{array}{c} CIFHM^{x}HM^{\left(x\right)}\left({Ç}_{1},{Ç}_{2},\ldots ,{Ç}_{n}\right)={Ç}^{-}. \end{array}$$

*CIFHM* ^*x*^ *HM* ^(*x*)^(*Ç*_1_, *Ç*_2_,…, *Ç*_*n*_) = *Ç*^+^.

From monotonicity property:

(24) $$\begin{array}{c} {Ç}^{-}\leq CIFHM^{x}\left({Ç}_{1},{Ç}_{2},\ldots ,{Ç}_{n}\right)\leq {Ç}^{+}. \end{array}$$

In a decision-making problem, the weights of all attributes and the experts sometimes matter. So we discuss the influence of weights on the HM operator in this section and develop the weighted HM operator as follows.

**Definition 12 .**

Let *Ç*_*j*_=(*r*_*μ*_*j*__(*x*)*e*^*iθ*_*μj*_(*x*)^, *s*_*νj*_(*x*)*e*^*iΦ*_*νj*_(*x*)^), *j*=1,2,…, *k* be the collection of CIFNs with weight vector *w*_*i*_=(*w*_1_, *w*_2_,…,*w*_*n*_)^*T*^,  *w*_*i*_ ∈ [0,1] and ∑_*i*=1_^*n*^*w*_*i*_=1. Then the CIFWHM operator is defined as follows:

(25) $$\begin{array}{c} {\text{CIFWHM}}^{\left(x\right)}\left({Ç}_{1},{Ç}_{2},\ldots ,{Ç}_{n}\right)=\frac{{⊕}_{1\leq i_{1}<\cdots <i_{x}\leq n}{\left({⊕}_{i=1}^{x}{\left({Ç}_{i_{j}}\right)}^{w_{i_{j}}}\right)}^{1/x}}{C_{n}^{x}}, \end{array}$$

**Theorem 5 .**

Let *Ç*_*j*_=(*r*_*μ*_*j*__(*x*)(*x*)*e*^2*πiθ*_*μj*_(*x*)^, *s*_*νj*_(*x*)*e*^2*πiΦ*_*νj*_(*x*)^), *j*=1,2,…, *k* be the collection of CIFNs. Then the aggregated value of the CIFWHM operator is also a CIFN such that

(26) $$\begin{array}{c} {\text{CIFWHM}}^{\left(x\right)}\left({Ç}_{1},{Ç}_{2},\ldots ,{Ç}_{n}\right)=\left(\left(\begin{array}{c} 1-{\left(\underset{1\leq i_{1}<\cdots <i_{x}\leq n}{\prod }\left(1-{\left(\overset{x}{\underset{j=1}{\prod }}r_{\mu_{i_{j}}}^{w_{i_{j}}}\left(x\right)\right)}^{1/x}\right)\right)}^{1/C_{n}^{x}}\cdot e^{2\pi i\left(1-{\left(\prod_{1\leq i_{1}<\cdots <i_{x}\leq n}\left(1-{\left(\prod_{j=1}^{x}\theta_{\mu_{i_{j}}}^{w_{i_{j}}}\left(x\right)\right)}^{1/x}\right)\right)}^{1/C_{n}^{x}}\right)},\\ {\left(\underset{1\leq i_{1}<\cdots <i_{x}\leq n}{\prod }\left(1-{\left(\overset{x}{\underset{j=1}{\prod }}{\left(1-s_{\nu_{i_{j}}}\left(x\right)\right)}^{w_{i_{j}}}\right)}^{1/x}\right)\right)}^{1/C_{n}^{x}}\cdot e^{2\pi i\left({\left(\prod_{1\leq i_{1}<\cdots <i_{x}\leq n}\left(1-{\left(\prod_{j=1}^{x}{\left(1-\Phi_{\nu_{i_{j}}}\left(x\right)\right)}^{w_{i_{j}}}\right)}^{1/x}\right)\right)}^{1/C_{n}^{x}}\right)} \end{array}\right)\right). \end{array}$$

**Proof**

(27) $$\begin{array}{c} {\left({Ç}_{j}\right)}^{w_{j}}={\left(r_{\mu_{i_{j}}}\left(x\right)\right)}^{w_{i_{j}}}e^{2\pi \text{i}{\left(\theta_{\mu_{i_{j}}}\left(x\right)\right)}^{w_{i_{j}}}},1-{\left(1-s_{\nu_{i_{j}}}\left(x\right)\right)}^{w_{i_{j}}}e^{2\pi i\left(1-{\left(1-\Phi_{\nu_{i_{j}}}\left(x\right)\right)}^{w_{i_{j}}}\right)},\\ \overset{x}{\underset{i=1}{⊕}}{\left({Ç}_{i_{j}}\right)}^{w_{i_{j}}}=\left(\begin{array}{c} \overset{x}{\underset{j=1}{\prod }}{\left(r_{\mu_{i_{j}}}\left(x\right)\right)}^{w_{i_{j}}}e^{2\pi \text{i}\left(\left(\prod_{j=1}^{x}{\left(\theta_{\mu_{i_{j}}}\left(x\right)\right)}^{w_{i_{j}}}\right)\right)},\\ 1-\left(\overset{x}{\underset{j=1}{\prod }}{\left(1-s_{\nu_{i_{j}}}\left(x\right)\right)}^{w_{i_{j}}}\right)e^{2\pi i\left(1-\left(\prod_{j=1}^{x}{\left(1-\Phi_{\nu_{i_{j}}}\left(x\right)\right)}^{w_{i_{j}}}\right)\right)} \end{array}\right),\\ {\left(\overset{x}{\underset{i=1}{⊕}}{\left({Ç}_{i_{j}}\right)}^{w_{i_{j}}}\right)}^{1/x}=\left(\begin{array}{c} {\left(\overset{x}{\underset{j=1}{\prod }}{\left(r_{\mu_{i_{j}}}\left(x\right)\right)}^{w_{i_{j}}}\right)}^{1/x}e^{2\pi \text{i}\left({\left(\left(\prod_{j=1}^{x}{\left(\theta_{\mu_{i_{j}}}\left(x\right)\right)}^{w_{i_{j}}}\right)\right)}^{1/x}\right)},\\ 1-{\left(\overset{x}{\underset{j=1}{\prod }}{\left(1-s_{\nu_{i_{j}}}\left(x\right)\right)}^{w_{i_{j}}}\right)}^{1/x}e^{2\pi i\left(1-{\left(\prod_{j=1}^{x}{\left(1-\Phi_{\nu_{i_{j}}}\left(x\right)\right)}^{w_{i_{j}}}\right)}^{1/x}\right)} \end{array}\right),\\ {⊕}_{1\leq i_{1}<,\ldots ,<i_{x}\leq n}{\left(\overset{x}{\underset{i=1}{⊕}}{\left({Ç}_{i_{j}}\right)}^{w_{i_{j}}}\right)}^{1/x}=\left(\begin{array}{c} 1-\underset{1\leq i_{1}<,\;\ldots ,\;<i_{x}\leq n}{\prod }\;\left(1-{\left(\overset{x}{\underset{j=1}{\prod }}{\left(r_{\mu_{i_{j}}}\left(x\right)\right)}^{w_{i_{j}}}\right)}^{1/x}\right).e^{2\pi i\left(1-\prod_{1\leq i_{1}<,\ldots ,<i_{x}\leq n}\left(1-{\left(\prod_{j=1}^{x}{\left(\theta_{\mu_{i_{j}}}\left(x\right)\right)}^{w_{i_{j}}}\right)}^{1/x}\right)\right)},\\ \underset{1\leq i_{1}<,\ldots ,<i_{x}\leq n}{\prod }\;\left(1-{\left(\overset{x}{\underset{j=1}{\prod }}{\left(1-s_{\nu_{i_{j}}}\left(x\right)\right)}^{w_{i_{j}}}\right)}^{1/x}\right).e^{2\Pi \text{i}\left(\prod_{1\leq i_{1}<,\ldots ,<i_{x}\leq n}\left(1-{\left(\prod_{j=1}^{x}{\left(1-\Phi_{\nu_{i_{j}}}\left(x\right)\right)}^{w_{i_{j}}}\right)}^{1/x}\right)\right)} \end{array}\right),\\ CIFWHM^{\left(x\right)}\left({Ç}_{1},{Ç}_{2},\ldots ,{Ç}_{n}\right)==\left(\begin{array}{c} 1-{\left(\underset{1\leq i_{1}<,\ldots ,<i_{x}\leq n}{\prod }\left(1-{\left(\overset{x}{\underset{j=1}{\prod }}r_{\mu_{i_{j}}}^{w_{i_{j}}}\left(x\right)\right)}^{1/x}\right)\right)}^{1/C_{n}^{x}}.e^{2\pi i\;\left(1-{\left(\prod_{1\leq i_{1}<,\ldots ,<i_{x}\leq n}\left(1-{\left(\prod_{j=1}^{x}\theta_{\mu_{i_{j}}}^{w_{i_{j}}}\left(x\right)\right)}^{1/x}\right)\right)}^{1/C_{n}^{x}}\right)},\\ {\left(\underset{1\leq i_{1}<,\ldots ,<i_{x}\leq n}{\prod }\left(1-{\left(\overset{x}{\underset{j=1}{\prod }}{\left(1-s_{\nu_{i_{j}}}\left(x\right)\right)}^{w_{i_{j}}}\right)}^{1/x}\right)\right)}^{1/C_{n}^{x}}.e^{2\pi i\;\left({\left(\prod_{1\leq i_{1}<,\ldots ,<i_{x}\leq n}\left(1-{\left(\prod_{j=1}^{x}{\left(1-\Phi_{\nu_{i_{j}}}\left(x\right)\right)}^{w_{i_{j}}}\right)}^{1/x}\right)\right)}^{1/C_{n}^{x}}\right)} \end{array}\right). \end{array}$$

Now, we have to show that is a CIFN.

(28) $$\begin{array}{c} r_{\mu }\left(x\right),s_{\nu }\left(x\right)\in \left[0,1\right],\theta_{\mu }\left(x\right),\Delta_{\nu }\left(x\right)\in \left[0,2\pi \right]. \end{array}$$

1. 0 ≤ *r*_*μ*_(*x*)+*s*_*ν*_(*x*) ≤ 1 and 0 ≤ *θ*_*μ*_(*x*)+Δ_*ν*_(*x*) ≤ 1

(29) $$\begin{array}{c} r_{\mu }\;\left(x\right)=1-{\left(\underset{1\leq i_{1}<\cdots <i_{x}\leq n}{\prod }\left(1-{\left(\overset{x}{\underset{j=1}{\prod }}r_{\mu_{i_{j}}}^{w_{i_{j}}}\left(x\right)\right)}^{1/x}\right)\right)}^{1/C_{n}^{x}},\\ \theta_{\mu }\left(x\right)=\;\left(1-{\left(\underset{1\leq i_{1}<\cdots <i_{x}\leq n}{\prod }\left(1-{\left(\overset{x}{\underset{j=1}{\prod }}\theta_{\mu_{i_{j}}}^{w_{i_{j}}}\left(x\right)\right)}^{1/x}\right)\right)}^{1/C_{n}^{x}}\right),\\ s_{\nu }\left(x\right)={\left(\underset{1\leq i_{1}<\cdots <i_{x}\leq n}{\prod }\left(1-{\left(\overset{x}{\underset{j=1}{\prod }}{\left(1-s_{\nu_{i_{j}}}\left(x\right)\right)}^{w_{i_{j}}}\right)}^{1/x}\right)\right)}^{1/C_{n}^{x}},\\ \Delta_{\nu }\left(x\right)=\left({\left(\underset{1\leq i_{1}<\cdots <i_{x}\leq n}{\prod }\left(1-{\left(\overset{x}{\underset{j=1}{\prod }}{\left(1-\Phi_{\nu_{i_{j}}}\left(x\right)\right)}^{w_{i_{j}}}\right)}^{1/x}\right)\right)}^{1/C_{n}^{x}}\right). \end{array}$$

We know that 0 ≤ *r*_*μ*_(*x*) ≤ 1 and 0 ≤ *θ*_*μ*_(*x*) ≤ 1 We have

(30) $$\begin{array}{c} 0\leq r_{\mu }\left(x\right)e^{2\pi i\theta_{\mu }\left(x\right)}\leq 1,\\ 0\leq \overset{x}{\underset{j=1}{\prod }}r_{\mu_{j}}\left(x\right)e^{2\pi i\left(\prod_{j=1}^{x}\theta_{\mu_{j}}\left(x\right)\right)}\leq 1,\\ 0\leq \overset{x}{\underset{j=1}{\prod }}{\left(r_{\mu_{j}}\left(x\right)\right)}^{w_{j}}e^{2\pi i\left(\prod_{j=1}^{x}{\left(\theta_{\mu_{j}}\left(x\right)\right)}^{w_{j}}\right)}\leq 1,\\ 0\leq 1-{\left(\overset{x}{\underset{j=1}{\prod }}{\left(r_{\mu_{j}}\left(x\right)\right)}^{w_{j}}\right)}^{1/x}e^{2\pi i\left(1-{\left(\prod_{j=1}^{x}{\left(\theta_{\mu_{j}}\left(x\right)\right)}^{w_{j}}\right)}^{1/x}\right)}\leq 1,\\ 0\leq \underset{1\leq i_{1}<\cdots <i_{x}\leq n}{\prod }\left(1-{\left(\overset{x}{\underset{j=1}{\prod }}{\left(r_{\mu_{j}}\left(x\right)\right)}^{w_{j}}\right)}^{1/x}\right)\cdot e^{2\pi i\left(\prod_{1\leq i_{1}<\cdots <i_{x}\leq n}\left(1-{\left(\prod_{j=1}^{x}{\left(\theta_{\mu_{j}}\left(x\right)\right)}^{w_{j}}\right)}^{1/x}\right)\right)}\leq 1,\\ 0\leq {\left(1-\underset{1\leq i_{1}<\cdots <i_{x}\leq n}{\prod }\left(1-{\left(\overset{x}{\underset{j=1}{\prod }}{\left(1-r_{\mu_{j}}\left(x\right)\right)}^{w_{j}}\right)}^{1/x}\right)\right)}^{1/C_{n}^{x}}\cdot e^{2\pi i\;\left({\left(1-\prod_{1\leq i_{1}<\cdots <i_{x}\leq n}\left(1-{\left(\prod_{j=1}^{x}{\left(1-\theta_{\nu_{j}}\left(x\right)\right)}^{w_{j}}\right)}^{1/x}\right)\right)}^{1/C_{n}^{x}}\right)}\leq 1. \end{array}$$

Similarly,

(31) $$\begin{array}{c} 0\leq s_{\nu }\left(x\right)e^{2\pi i_{\nu }\left(x\right)}\leq 1. \end{array}$$

Since 0 ≤ *r*_*μ*_(*x*)*e*^2*πiθ*_*μ*_(*x*)^ ≤ 1 and 0 ≤ *s*_*ν*_(*x*)*e*^2*πi*_*Φν*_(*x*)^ ≤ 1 so we have

(32) $$\begin{array}{c} 0\leq r_{\mu }\left(x\right)e^{2\pi i\theta_{\mu }\left(x\right)}+s_{\nu }\left(x\right)e^{2\pi i_{\nu }\left(x\right)}\leq 1. \end{array}$$

We gave Example 2 to support Definition 11 and aggregate the values of some CIFNs by utilizing the CIFWHM operator.

**Example 2 .**

Let *Ç*_1_=0.45*e*^2*πi*(0.3)^, 0.62*e*^2*πi*(0.41)^, *Ç*_2_=0.2*e*^2*πi*(0.7)^, 0.52*e*^2*πi*(0.6)^, *Ç*_3_=0.5*e*^2*πi*(0.6)^, 0.2*e*^2*πi*(0.91)^, *Ç*_4_=0.7*e*^2*πi*(0.8)^, 0.42*e*^2*πi*(0.15)^ be the CIFNs with weight vector *w*=(0.3, 0.1, 0.4, 0.2). Then we use the proposed CIFWHM operator to aggregate the given CIFNs. Suppose that *x*=2.

(33) $$\begin{array}{c} {\text{CIFWHM}}^{\left(x\right)}\left({Ç}_{1},{Ç}_{2},\ldots ,{Ç}_{n}\right)=\left(\begin{array}{c} 1-{\left(\underset{1\leq i_{1}<\cdots <i_{x}\leq n}{\prod }\left(1-{\left(\overset{x}{\underset{j=1}{\prod }}r_{\mu_{i_{j}}}^{w_{i_{j}}}\left(x\right)\right)}^{1/x}\right)\right)}^{1/C_{n}^{x}}\cdot e^{2\pi i\;\left(1-{\left(\prod_{1\leq i_{1}<\cdots <i_{x}\leq n}\left(1-{\left(\prod_{j=1}^{x}\theta_{\mu_{i_{j}}}^{w_{i_{j}}}\left(x\right)\right)}^{1/x}\right)\right)}^{1/C_{n}^{x}}\right)},\\ {\left(\underset{1\leq i_{1}<\cdots <i_{x}\leq n}{\prod }\left(1-{\left(\overset{x}{\underset{j=1}{\prod }}{\left(1-s_{\nu_{i_{j}}}\left(x\right)\right)}^{w_{i_{j}}}\right)}^{1/x}\right)\right)}^{1/C_{n}^{x}}\cdot e^{2\pi i\;\left({\left(\prod_{1\leq i_{1}<\cdots <i_{x}\leq n}\left(1-{\left(\prod_{j=1}^{x}{\left(1-\Phi_{\nu_{i_{j}}}\left(x\right)\right)}^{w_{i_{j}}}\right)}^{1/x}\right)\right)}^{1/C_{n}^{x}}\right)} \end{array}\right)=\left(0.8e^{2\pi i\left(0.9\right)},0.1e^{2\pi i\left(0.2\right)}\right). \end{array}$$

**Theorem 6 (Idempotency Property).**

Let *Ç*_*j*_=(*r*_*μ*_*j*__(*x*)*e*^*iθ*_*μ*_*j*__(*x*)^, *s*_*ν*_*j*__(*x*)*e*^*iΦ*_*νjj*_(*x*)^),  *j*=1,2,…, *k* be the collection of all identical values of CIFNs. Then

(34) $$\begin{array}{c} {\text{CIFWHM}}^{x}\left({Ç}_{1},{Ç}_{2},\ldots ,{Ç}_{n}\right)=Ç. \end{array}$$

**Proof**

Similar to Theorem 2, we can easily prove Theorem 6.

**Theorem 7 (Monotonicity Property).**

Let *Ç*_*j*_=(*r*_*μ*_*j*__(*x*)*e*^*iθ*_*μ*_*j*__(*x*)^, *s*_*ν*_*j*__(*x*)*e*^*iΦ*_*ν*_*j*__(*x*)^),  *j*=1,2,…, *k* and *D*_*j*_=(*g*_*μ*_*j*__(*x*)*e*^*iα*_*μ*_*j*__(*x*)^, *h*_*ν*_*j*__(*x*)*e*^*iβ*_*ν*_*j*__(*x*)^), *j*=1,2,…, *k* be two sets of CIFNs. Then *r*_*μ*_*j*__(*x*) < *g*_*μ*_*j*__(*x*), *θ*_*μ*_ _*j*_(*x*) < *α*_*μ*_ _*j*_(*x*) and *s*_*ν*_*j*__(*x*) > *h*_*ν*_*j*__(*x*),  *Φ*_*ν*_*j*__(*x*) > *β*_*ν*_*j*__(*x*). Then

(35) $$\begin{array}{c} {\text{CIFWHM}}^{x}\left({Ç}_{1},{Ç}_{2},\ldots ,{Ç}_{n}\right)\leq {\text{CIFWHM}}^{x}\left(D_{1},D_{2},\ldots ,D_{n}\right). \end{array}$$

**Proof**

Similar to Theorem 3, we can easily prove Theorem 7.

**Theorem 8 (Boundedness Property).**

Let *Ç*_*j*_=(*r*_*μ*_*j*__(*x*)*e*^*iθ*_*μ*_*j*__(*x*)^, *s*_*ν*_*j*__(*x*)*e*^*iΦ*_*ν*_*j*__(*x*)^),  *j*=1,2,…, *k* be the collection of CIFNs. If

(36) $$\begin{array}{c} {Ç}_{j}^{-}=\min\left({Ç}_{1},{Ç}_{2},{Ç}_{3},\ldots ,{Ç}_{n}\right),\\ {Ç}_{j}^{+}=\max\left({Ç}_{1},{Ç}_{2},{Ç}_{3},\ldots ,{Ç}_{n}\right), \end{array}$$

then

(37) $$\begin{array}{c} {Ç}^{-}\leq {\text{CIFWHM}}^{x}\left({Ç}_{1},{Ç}_{2},\ldots ,{Ç}_{n}\right)\leq {Ç}^{+}. \end{array}$$

From boundedness property:

(38) $$\begin{array}{c} {\text{CIFWHM}}^{x}\left({Ç}_{1},{Ç}_{2},\ldots ,{Ç}_{n}\right)={Ç}^{-}. \end{array}$$

(39) $$\begin{array}{c} {\text{CIFWHM}}^{x}\left({Ç}_{1},{Ç}_{2},\ldots ,{Ç}_{n}\right)={Ç}^{+} \end{array}$$

CIFWHM^*x*^(*Ç*_1_, *Ç*_2_,…, *Ç*_*n*_) = *Ç*^+^.

From monotonicity property:

(40) $$\begin{array}{c} {Ç}^{-}\leq {\text{CIFWHM}}^{x}\left({Ç}_{1},{Ç}_{2},\ldots ,{Ç}_{n}\right)\leq {Ç}^{+}. \end{array}$$

**Proof**

From Theorem 5, we have

(41) $$\begin{array}{c} {\text{CIFWHM}}^{\left(x\right)}\left({Ç}_{1}^{-},{Ç}_{2}^{-},\ldots ,{Ç}_{n}^{-}\right)=\left(\begin{array}{c} 1-{\left(\underset{1\leq i_{1}<\cdots <i_{x}\leq n}{\prod }\left(1-{\left(\overset{x}{\underset{j=1}{\prod }}\min\left(r_{\mu_{j}}^{w_{i_{j}}}\left(x\right)\right)\right)}^{1/x}\right)\right)}^{1/C_{n}^{x}}\cdot e^{2\pi i\;\left(1-{\left(\prod_{1\leq i_{1}<\cdots <i_{x}\leq n}\left(1-{\left(\prod_{j=1}^{x}\min\left(\theta_{\mu_{j}}^{w_{i_{j}}}\left(x\right)\right)\right)}^{1/x}\right)\right)}^{1/C_{n}^{x}}\right)},\\ {\left(\underset{1\leq i_{1}<\cdots <i_{x}\leq n}{\prod }\left(1-{\left(\overset{x}{\underset{j=1}{\prod }}{\left(1-\max\left(s_{\nu_{j}}\left(x\right)\right)\right)}^{w_{i_{j}}}\right)}^{1/x}\right)\right)}^{1/C_{n}^{x}}\cdot e^{2\pi i\;\left({\left(\prod_{1\leq i_{1}<\cdots <i_{x}\leq n}\left(1-{\left(\prod_{j=1}^{x}{\left(1-\max\left(\Delta_{\nu_{j}}\left(x\right)\right)\right)}^{w_{i_{j}}}\right)}^{1/x}\right)\right)}^{1/C_{n}^{x}}\right)} \end{array}\right),\\ {\text{CIFWHM}}^{\left(x\right)}\left({Ç}_{1}^{+},{Ç}_{2}^{+},\ldots ,{Ç}_{n}^{+}\right)=\left(\begin{array}{c} 1-{\left(\underset{1\leq i_{1}<\cdots <i_{x}\leq n}{\prod }\left(1-{\left(\overset{x}{\underset{j=1}{\prod }}\max\left(r_{\mu_{j}}^{w_{i_{j}}}\left(x\right)\right)\right)}^{1/x}\right)\right)}^{1/C_{n}^{x}}\cdot e^{2\pi i\;\left(1-{\left(\prod_{1\leq i_{1}<\cdots <i_{x}\leq n}\left(1-{\left(\prod_{j=1}^{x}\max\left(\theta_{\mu_{j}}^{w_{i_{j}}}\left(x\right)\right)\right)}^{1/x}\right)\right)}^{1/C_{n}^{x}}\right)},\\ {\left(\underset{1\leq i_{1}<\cdots <i_{x}\leq n}{\prod }\left(1-{\left(\overset{x}{\underset{j=1}{\prod }}{\left(1-\min\left(s_{\nu_{j}}\left(x\right)\right)\right)}^{w_{i_{j}}}\right)}^{1/x}\right)\right)}^{1/C_{n}^{x}}\cdot e^{2\pi i\;\left({\left(\prod_{1\leq i_{1}<\cdots <i_{x}\leq n}\left(1-{\left(\prod_{j=1}^{x}{\left(1-\min\left(\Delta_{\nu_{j}}\left(x\right)\right)\right)}^{w_{i_{j}}}\right)}^{1/x}\right)\right)}^{1/C_{n}^{x}}\right)} \end{array}\right). \end{array}$$

From property 4, we have

(42) $$\begin{array}{c} {Ç}^{-}\leq {\text{CIFWHM}}^{x}\left({Ç}_{1},{Ç}_{2},\ldots ,{Ç}_{n}\right)\leq {Ç}^{+}. \end{array}$$

## 5. The Dual Hamy Mean Operator

In this section, we use the idea of the DHM operator in the framework of CIFSs. We prove the validity of the proposed AO. We also give a numerical example to support the proposed CIFDHM operator.

**Definition 13 .**

Let *Ç*_*j*_=(*r*_*μ*_*j*__(*x*)*e*^*iθ*_*μ*_*j*__^, *s*_*ν*_*j*__(*x*)*e*^*iΦ*_*ν*_*j*__^),  *j*=1,2,…, *k* be the collection of CIFNs. Then CIFDHM operator is defined as follows:

(43) $$\begin{array}{c} {\text{CIFDHM}}^{\left(x\right)}\left({Ç}_{1},{Ç}_{2},\ldots ,{Ç}_{n}\right)={\left(\underset{1\leq i_{1}<\cdots <i_{x}\leq n}{\prod }\left(\frac{\sum_{j=1}^{x}{Ç}_{j}}{x}\right)\right)}^{1/C_{n}^{x}}. \end{array}$$

**Theorem 9 .**

Let *Ç*_*j*_=(*r*_*μ*_*j*__(*x*)*e*^*iθ*_*μ*_*j*__^, *s*_*ν*_*j*__(*x*)*e*^*i*_*Φν*_*j*__^),  *j*=1,2,…, *k* be the collection of CIFNs. Then CIFDHM operator is defined as follows:

(44) $$\begin{array}{c} {\text{CIFDHM}}^{\left(x\right)}\left({Ç}_{1},{Ç}_{2},\ldots ,{Ç}_{n}\right)={\left(\underset{1\leq i_{1}<\cdots <i_{x}\leq n}{\prod }\left(\frac{\sum_{j=1}^{x}{Ç}_{j}}{x}\right)\right)}^{1/C_{n}^{x}}. \end{array}$$

**Proof**

The proof is analogous to the proof of Theorem 1.

**Remark 2 .**

The CIFDHM operator also satisfies the basic properties of aggregation as discussed in Theorems 2–4.

Now we will elaborate on the concepts of DHM operator in the framework of CIFSs by keeping the weight under observation.

**Definition 14 .**

Let *Ç*_*j*_=(*r*_*μ*_*j*__(*x*)*e*^*iθ*_*μ*_*j*__(*x*)^, *s*_*ν*_*j*__(*x*)*e*^*iΦ*_*ν*_*j*__(*x*)^), *j*=1,2,…, *k* be the collection of CIFNs with weight vector *w*_*i*_=(*w*_1_, *w*_2_,…,*w*_*n*_)^*T*^,  *w*_*i*_ ∈ [0,1], and ∑_*i*=1_^*n*^*w*_*i*_=1. Then the CIFWDHM operator is defined as follows:

(45) $$\begin{array}{c} {\text{CIFWDHM}}^{\left(x\right)}\left({Ç}_{1},{Ç}_{2},\ldots ,{Ç}_{n}\right)={\left(\Delta_{1\leq i_{1}<\cdots <i_{x}\leq n}\left(\frac{{⊕}_{i=1}^{x}w_{i_{j}}{Ç}_{i_{j}}}{x}\right)\right)}^{1/C_{n}^{x}}. \end{array}$$

**Theorem 13 .**

Let *Ç*_*j*_=(*r*_*μ*_*j*__(*x*)*e*^*iθ*_*μ*_*j*__(*x*)^, *s*_*ν*_*j*__(*x*)*e*^*iΦ*_*ν*_*j*__(*x*)^), *j*=1,2,…, *k* be the collection of CIFNs. Then the aggregated value of the CIFWDHM operator is also a CIFN and is given by

(46) $$\begin{array}{c} {\text{CIFWDHM}}^{\left(x\right)}\left({Ç}_{1},{Ç}_{2},\ldots ,{Ç}_{n}\right)=\left(\begin{array}{c} {\left(\underset{1\leq i_{1}<\cdots <i_{x}\leq n}{\prod }\left(1-{\left(\overset{x}{\underset{j=1}{\prod }}{\left(1-r_{\mu_{i_{j}}}\left(x\right)\right)}^{w_{i_{j}}}\right)}^{1/x}\right)\right)}^{1/C_{n}^{x}}\cdot e^{2\pi i\left({\left(\prod_{1\leq i_{1}<\cdots <i_{x}\leq n}\left(1-{\left(\prod_{j=1}^{x}{\left(1-\theta_{\mu_{i_{j}}}\left(x\right)\right)}^{w_{i_{j}}}\right)}^{1/x}\right)\right)}^{1/C_{n}^{x}}\right)}\\ 1-{\left(\underset{1\leq i_{1}<\cdots <i_{x}\leq n}{\prod }\left(1-{\left(\overset{x}{\underset{j=1}{\prod }}{\left(s_{\nu_{i_{j}}}\left(x\right)\right)}^{w_{i_{j}}}\right)}^{1/x}\right)\right)}^{1/C_{n}^{x}}\cdot e^{2\pi i\;\left(1-{\left(\prod_{1\leq i_{1}<\cdots <i_{x}\leq n}\left(1-{\left(\prod_{j=1}^{x}{\left(\Delta_{\nu_{j}}\left(x\right)\right)}^{w_{i_{j}}}\right)}^{1/x}\right)\right)}^{1/C_{n}^{x}}\right)} \end{array}\right). \end{array}$$

**Proof**

The proof is similar to the proof of Theorem 5.

We gave Example 3 to support Definition 13 and aggregate some CIFNs by utilizing the proposed CIFWDHM operators.

**Example 3 .**

Let *Ç*_1_=0.55*e*^2*πi*(0.45)^, 0.70*e*^2*πi*(0.85)^, *Ç*_2_=0.40*e*^2*πi*(0.70)^, 0.82*e*^2*πi*(0.77)^, *Ç*_3_=0.60*e*^2*πi*(0.40)^,  0.72*e*^2*πi*(0.60)^, *Ç*_4_=0.50*e*^2*πi*(0.75)^, 0.52*e*^2*πi*(0.35)^ be the CIFNs with the weight vector of the attributes be *w*=(0.4, 0.2, 0.3, 0.1). Then we use the proposed CIFWDHM operator to investigate the CIFN. Suppose that *x*=2.

(47) $$\begin{array}{c} {\text{CIFWDHM}}^{\left(x\right)}\left({Ç}_{1},{Ç}_{2},\ldots ,{Ç}_{n}\right)=\left(\begin{array}{c} {\left(\underset{1\leq i_{1}<,\ldots ,<i_{x}\leq n}{\prod }\left(1-{\left(\overset{x}{\underset{j=1}{\prod }}{\left(1-r_{\mu_{i_{j}}}\left(x\right)\right)}^{w_{i_{j}}}\right)}^{1/x}\right)\right)}^{1/C_{n}^{x}}.e^{2\pi i\;\left({\left(\prod_{1\leq i_{1}<,\ldots ,<i_{x}\leq n}\left(1-{\left(\prod_{j=1}^{x}{\left(1-\theta_{\mu_{i_{j}}}\left(x\right)\right)}^{w_{i_{j}}}\right)}^{1/x}\right)|\;\right)}^{1/C\left(x/n\right)}\right)}\\ 1-{\left(\underset{1\leq i_{1}<,\ldots ,<i_{x}\leq n}{\prod }\left(1-{\left(\overset{x}{\underset{j=1}{\prod }}{\left(s_{\nu_{i_{j}}}\left(x\right)\right)}^{w_{i_{j}}}\right)}^{1/x}\right)\right)}^{1/C\left(x/n\right)}.e^{2\pi i\left(1-{\left(\prod_{1\leq i_{1}<,\ldots ,<i_{x}\leq n}\left(1-{\left(\prod_{j=1}^{x}{\left({\left(x\right)}_{\nu_{i_{j}}}\right)}^{w_{i_{j}}}\right)}^{1/x}\right)\right)}^{1/Cx/n}\right)} \end{array}\right)=\left(0.16e^{2\pi i\left(0.17\right)},0.92e^{2\pi i\left(0.92\right)}\right). \end{array}$$

**Remark 3 .**

The CIFWDHM operator also satisfies the basic properties of aggregation as discussed in Theorems 2, 3, and 8.

## 6. Application

A CIFS is an extension of an IFSs in which the sum of TD and FD lies on interval [0,1]; each TD and FD has two aspects: amplitude terms and phase terms. In this section, we developed a MADM method based on CIFWHM and CIFWDHM operators to solve a problem involving the development of the tourism industry. First, we proposed the MADM algorithm, and then we present a comprehensive example to utilize the proposed algorithm.

### 6.1. Algorithm

In this subsection, we describe the steps of the algorithm as follows:

  Step 1: Information are collected from the decision-maker about the tourism industry (about the finite number of alternatives based on attributes). All information are in the form of CIFNSs.
  Step 2: This step involves the utilization of the proposed CIFWHM and CIFWDHM operator to aggregate the CIF information depicted in the decision matrix given in step 1.
  Step 3: This step is about the analysis of the aggregated information based on score values of CIFNs by using Definition 3.
  Step 4: In this step, we investigate the score values of all aggregated information for ordering and ranking the attributes.

**Example 4 .**

The average increase in the economy induces ever-greater competitive surroundings for tourism enterprises. The essential competition of the tourism industry depends on the tour destination. In the tourism environment, to assure the regular development of a visitor vacation spot, it is essential to take measures to increase tourism vacation spots to enhance competitiveness. The vital role of the tourism industry is to analyze and evaluate the vacation spot competitiveness, which can display the attraction strength of a vacation spot and imply the direction for the efficient allocation of assets. Therefore, it is a crucial manner for determining the vacation spot development mode and route to analyze the important elements of tourism destination and extract qualitative and quantitative assessment index, which can uphold long-term competitive benefit and the nonstop improvement of vacation spot. This is a problem of interest, and we try to investigate this problem using our proposed AOs and adopt the tourism destination problem from [25].

Consider five possible tourist destinations *A*_*i*_(*i*=1,2,3,4,5) that have to be evaluated. These five destinations are to be examined based on four attributes where *G*_1_: is the attractiveness of tourism sources, *G*_2_ is the infrastructure and development of the tourism industry, *G*_3_ is supporting force of the tourism environment, and *G*_4_ is the tourist demand. The five possible vacationer locations are to be assessed with IVIFNs that are weighting vectors *w*=(0.4, 0.2, 0.1, 0.3) as shown in Table 1. Then, we use the approach developed to decide on the best tourist destinations.

  Step 1: The decision matrix containing the information about the five alternatives by anonymous decision-makers is provided in Table 1.
  Step 2: Aggregate CIFNs are shown in the decision matrix in Table 1, according to the given attributes by the decision-maker utilizing the proposed AOs of CIFWHM and CIFWDHM at the parameter *x*=3. All aggregated results are shown in Table 2.
  Step 3: By utilizing Definition 3, we obtained the score values of the consequences shown in Table 2, and the results are shown in Table 3.

In Table 4, we listed the order of the tourist destinations based on the score value obtained in Table 3. We observed that the best destination came out to be *A*_3_ by using the CIFWHM operator and *A*_1_ by using the CIFWDHM operator at the parameter of *x*=3. Consider Table 4 for further comprehensive orderings.

**Figure 1:**
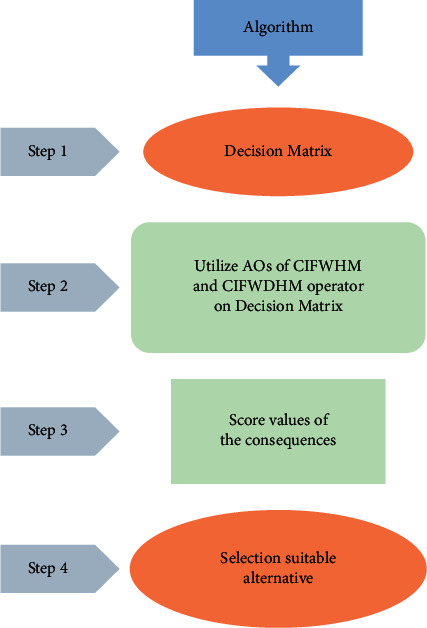
The flowchart of the algorithm. The steps in the flow chart are presented in Example 4.

Another view of the results of Tables 3 and 4 can be seen in Figure 1 where the results obtained by using CIFWHM operators are described in the first bar graph, while the results obtained by using CIFWDHM operators are described using the second bar graph in orange color. Results using both the HM operators can be seen explicitly.

### 6.2. Influence Study

In this subsection, we see the impact of parameter *x* on the results. We see the effect on ordering and ranking of the results by the variation of the parameter *x*=1,2,3,4 obtained by using CIFWHM and CIFWDHM operators. We listed the analysis of the results after varying the parameter *x* in Tables 5 and 6.

We analyzed *A*_3_ as the best tourist destination when we take *x*=1,2,3,4 in the case of the CIFWDHM operator also. Variation of *x* is studied in both cases, and we reached the following results:

1. Variation of *x* does not create any impact on ranking results.
2. Variation of *x* may have an impact in some other cases depending on the data we dealt with must be analyzed each time in each case.

## 7. Comparative Analysis

In this section, we aim to analyze the aggregated results obtained using the CIFHM operators with the aggregated results obtained using the AOs of Akram et al. [10], Wang et al. [42], Wu et al. [24], Li and Wei [37], Garg et al. [39], Ali et al. [43], and Fan et al. [44]. The comparison results can be seen in Table 7.

**Figure 2:**
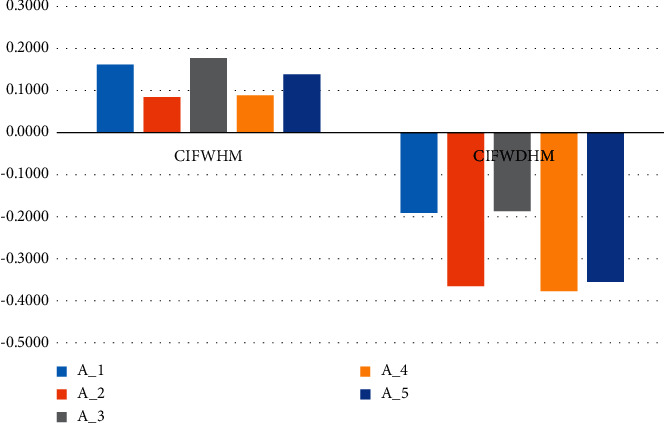
Score values of tourist destinations.

From Table 7, it is observed that the previously existing AOs in different fuzzy frameworks such as Wang et al. [42], Wu et al. [24], Li and Wei [37], and Fan et al. [44] cannot be applied to the information that is discussed in the decision matrix in CIF environment given in Table 1. Further, the results obtained using Garg et al. [39] and Akram et al. [10](by using CIFHWA operator) give us *A*_3_ is the suitable destination. Moreover, CIFHWG operator by Akram et al. [10] and CIFMSM operator by Ali et al. [43] gave *A*_5_ and *A*_1_ as suitable destinations, respectively. The only reason for having different results is that the operators discussed in [10, 39, 43] do not consider the relationship of the information being used, while our proposed HM operators take into account this factor and hence gave us reliable results. A geometrical view of comparison results is depicted in Figure 2 followed by the advantages of the proposed work.

The advantages of the proposed work are as follows:

1. CIFHM and CIFDHM operators are the generalizations of the results discussed in [39, 40] and so on
2. CIFWHM and CIFWDHM operators deal with such information that has two aspects of the TD and FD denoted by amplitude term and phase term

## 8. Conclusion

In this paper, we utilized the idea of HM operators to elaborate the CIFSs in the framework of CIFHM operators to find the reliability of CIFSs. A CIFS has two aspects: TD and FD; TD has also two aspects amplitude terms and phase terms; and similarly, FD has two parts: amplitude terms and phase terms of FD that carried the more flexible information. The main contribution and key factors in this paper are as follows:

1. We proposed CIFHM and CIFDHM operators based on CIFSs
2. We also investigated the basic properties of the proposed work in the form of idempotency, monotonicity, and boundedness
3. We elaborated the concepts of the DHM operator in the framework of CIFSs by keeping the weight under observation
4. We analyzed the consequences of the proposed work through application and numerical examples to illustrate the CIFSs.

## Figures and Tables

**Figure 3:**
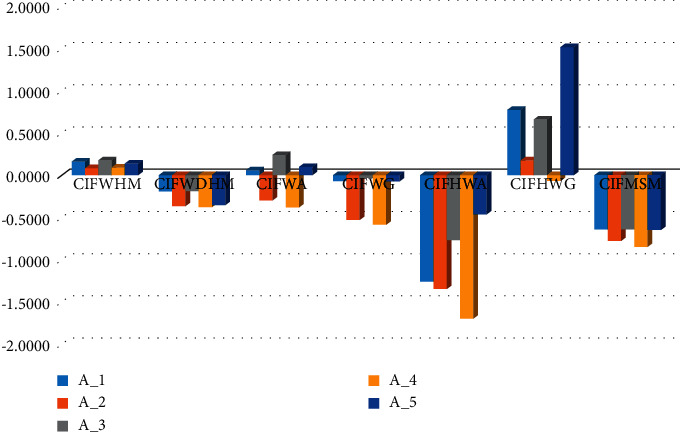
Comparison of score values of tourist destinations.

**Table 1:** Tourist destinations decision matrix.

	*A*_1_	*A*_2_	*A*_3_	*A*_4_	*A*_5_
*G*_1_	0.5*e*^2*πi*(0.3)^, 0.35*e*^2*πi*(0.4)^	0.3*e*^2*πi*(0.2)^, 0.6*e*^2*πi*(0.4)^	0.6*e*^2*πi*(0.2)^, 0.09*e*^2*πi*(0.3)^	0.1*e*^2*πi*(0.4)^, 0.4*e*^2*πi*(0.55)^	0.2*e*^2*πi*(0.5)^, 0.6*e*^2*πi*(0.33)^
*G*_2_	0.4*e*^2*πi*(0.2)^, 0.45*e*^2*πi*(0.4)^	0.1*e*^2*πi*(0.4)^, 0.7*e*^2*πi*(0.5)^	0.3*e*^2*πi*(0.3)^, 0.65*e*^2*πi*(0.4)^	0.3*e*^2*πi*(0.2)^, 0.6*e*^2*πi*(0.3)^	0.1*e*^2*πi*(0.5)^, 0.4*e*^2*πi*(0.18)^
*G*_3_	0.2*e*^2*πi*(0.5)^, 0.6*e*^2*πi*(0.39)^	0.1*e*^2*πi*(0.4)^, 0.5*e*^2*πi*(0.18)^	0.4*e*^2*πi*(0.4)^, 0.55*e*^2*πi*(0.5)^	0.2*e*^2*πi*(0.2)^, 0.6*e*^2*πi*(0.5)^	0.4*e*^2*πi*(0.2)^, 0.38*e*^2*πi*(0.4)^
*G*_4_	0.5*e*^2*πi*(0.3)^, 0.08*e*^2*πi*(0.4)^	0.3*e*^2*πi*(0.3)^, 0.35*e*^2*πi*(0.15)^	0.4*e*^2*πi*(0.3)^, 0.08*e*^2*πi*(0.25)^	0.4*e*^2*πi*(0.2)^, 0.45*e*^2*πi*(0.3)^	0.1*e*^2*πi*(0.5)^, 0.4*e*^2*πi*(0.28)^

**Table 2:** Aggregated values computed by using the CIFWHM and CIFWDHM operators.

	CIFWHM	CIFWDHM
*A*_1_	0.9541*e*^2*πi*(0.9247)^, 0.7610*e*^2*πi*(0.7948)^	0.8220*e*^2*πi*(0.7431)^, 0.9083*e*^2*πi*(0.9518)^
*A*_2_	0.9067*e*^2*πi*(0.9189)^, 0.8547*e*^2*πi*(0.8020)^	0.6831*e*^2*πi*(0.7638)^, 0.9635*e*^2*πi*(0.9332)^
*A*_3_	0.9629*e*^2*πi*(0.9093)^, 0.7346*e*^2*πi*(0.7838)^	0.8173*e*^2*πi*(0.7391)^, 0.9110*e*^2*πi*(0.9258)^
*A*_4_	0.9144*e*^2*πi*(0.9023)^, 0.8193*e*^2*πi*(0.8203)^	0.6784*e*^2*πi*(0.7417)^, 0.9596*e*^2*πi*(0.9328)^
*A*_5_	0.8886*e*^2*πi*(0.9528)^, 0.8241*e*^2*πi*(0.7399)^	0.6391*e*^2*πi*(0.8455)^, 0.9668*e*^2*πi*(0.8996)^

**Table 3:** Score values of tourist destinations.

	CIFWHM	CIFWDHM
*A*_1_	0.1615	−0.1907
*A*_2_	0.0844	−0.3650
*A*_3_	0.1769	−0.1870
*A*_4_	0.0886	−0.3767
*A*_5_	0.1387	−0.3548

**Table 4:** Order of tourist destinations.

Order						
CIFWHM	0.2482	0.2138	0.1082	0.0983	0.0706	*A*_3_ > *A*_1_ > *A*_5_ > *A*_4_ > *A*_2_
CIFWDHM	−0.1195	−0.1233	−0.3073	−0.3116	−0.3363	*A*_3_ > *A*_1_ > *A*_5_ > *A*_2_ > *A*_4_

**Table 5:** Results and order of the CIFWHM operator.

	Š(*A*_1_)	Š(*A*_2_)	Š(*A*_3_)	Š(*A*_4_)	Š(*A*_5_)	Order
*x*=1	−0.1735	−0.2896	−0.1649	−0.2950	−0.2286	*A*_3_ > *A*_1_ > *A*_4_ > *A*_2_ > *A*_5_
*x*=2	−0.1096	−0.2010	−0.1006	−0.2041	−0.1488	*A*_3_ > *A*_1_ > *A*_4_ > *A*_5_ > *A*_2_
*x*=3	0.1615	0.0844	0.1769	0.0886	0.1387	*A*_3_ > *A*_1_ > *A*_4_ > *A*_5_ > *A*_2_
*x*=4	0.7093	0.6172	0.7367	0.6373	0.6971	*A*_3_ > *A*_1_ > *A*_5_ > *A*_4_ > *A*_2_

The authors analyzed that *A*_3_ is the best tourist destination when the authors take *x*=1,2,3,4 in the case of the CIFWHM operator.

**Table 6:** Results and order of the CIFWDHM operator.

	Š(*A*_1_)	Š(*A*_2_)	Š(*A*_3_)	Š(*A*_4_)	Š(*A*_5_)	Order
*x*=1	0.3494	0.1029	0.3709	0.1044	0.1214	*A*_3_ > *A*_1_ > *A*_5_ > *A*_4_ > *A*_2_
*x*=2	0.2344	0.0404	0.2535	0.0402	−0.0577	*A*_3_ > *A*_1_ > *A*_2_ > *A*_4_ > *A*_5_
*x*=3	−0.1907	−0.3650	−0.1870	−0.3767	−0.3548	*A*_3_ > *A*_1_ > *A*_5_ > *A*_2_ > *A*_4_
*x*=4	−1.0137	−1.1977	−1.0252	−1.2181	−1.1644	*A*_3_ > *A*_1_ > *A*_5_ > *A*_2_ > *A*_4_

**Table 7:** The results and order of the CIFWDHM operator.

Operator	Environment	Results
CIFWHM operator (current work)	CIFSs	*A*_3_ > *A*_1_ > *A*_4_ > *A*_5_ > *A*_2_
CIFWDHM operator (current work)	CIFSs	*A*_3_ > *A*_1_ > *A*_5_ > *A*_2_ > *A*_4_
Garg et al. [39], CIFWA	CIFSs	*A*_3_ > *A*_5_ > *A*_1_ > *A*_2_ > *A*_4_
Garg et al. [39], CIFWG	CIFSs	*A*_3_ > *A*_1_ > *A*_5_ > *A*_2_ > *A*_4_
Akram et al. [10], CIFHWA	CIFSs	*A*_3_ > *A*_5_ > *A*_1_ > *A*_2_ > *A*_4_
Akram et al. [10], CIFHWG	CIFSs	*A*_5_ > *A*_1_ > *A*_3_ > *A*_2_ > *A*_4_
Ali et al. [43], CIFMSM	CIVIFSs	*A*_1_ > *A*_3_ > *A*_5_ > *A*_2_ > *A*_4_
Wang et al. [42]	CIVIFSs	Failed
Wu et al. [24]	IVIFSs	Failed
Wu et al. [24]	IVIFSs	Failed
Li and Wei [37]	PyFSs	Failed
Fan et al. [44]	IFSs	Failed

## Data Availability

The data used to support the findings of this study are available from the corresponding author upon request.

## References

[1] Zadeth L. A. (1965). *‘Fuzzy sets’, Information and control*.

[2] Atanassov K. T. (1999). Intuitionistic fuzzy sets. *Intuitionistic Fuzzy Sets*.

[3] Ramot D., Friedman M., Langholz G., Kandel A. (2003). Complex fuzzy logic. *IEEE Transactions on Fuzzy Systems*.

[4] Bajaj R. K. (2014). On complex intuitionistic fuzzy soft sets with distance measures and entropies. *Journal of Mathematics*.

[5] Nguyen H. T., Kandel A., Kreinovich V. Complex fuzzy sets: towards new foundations.

[6] Alkouri J. S., Salleh A. R. (2012). Complex intuitionistic fuzzy sets. *AIP Conference Proceedings*.

[7] Gulzar M., Mateen M. H., Alghazzawi D., Kausar N. (2020). A novel applications of complex intuitionistic fuzzy sets in group theory. *IEEE Access*.

[8] Rajareega S., Vimala J., Preethi D. (2020). Complex intuitionistic fuzzy soft lattice ordered group and its weighted distance measures. *Mathematics*.

[9] Khalaf M. M., Alharbi S. O., Chammam W. (2019). Similarity measures between temporal complex intuitionistic fuzzy sets and application in pattern recognition and medical diagnosis. *Discrete Dynamics in Nature and Society*.

[10] Akram M., Peng X., Sattar A. (2021). A new decision-making model using complex intuitionistic fuzzy Hamacher aggregation operators. *Soft Computing*.

[11] Rani D. (2020). Generalized geometric aggregation operators based on t-norm operations for complex intuitionistic fuzzy sets and their application to decision-making. *Cognitive Computation*.

[12] Salleh A., Ahmad A. (2017). Complex intuitionistic fuzzy normal subgroup. *International Journal of Pure and Apllied Mathematics*.

[13] Yu D. (2014). Intuitionistic fuzzy information aggregation under confidence levels. *Applied Soft Computing*.

[14] Liu X. (2012). Intuitionistic fuzzy information aggregation using Einstein operations. *IEEE Transactions on Fuzzy Systems*.

[15] Riaz M., Athar Farid H., Kalsoom H., Pamučar D., Chu Y.-M. (2020). A robust q-Rung orthopair fuzzy Einstein prioritized aggregation operators with application towards MCGDM. *Symmetry*.

[16] Hussain A., Ullah K., Alshahrani M. N., Yang M.-S., Pamucar D. (2022). Novel aczel-alsina operators for pythagorean fuzzy sets with application in multi-attribute decision making. *Symmetry*.

[17] Ashraf A., Ullah K., Ullah A., Hussain M., Bari M. (2022). Interval-valued picture fuzzy maclaurin symmetric mean operator with application in multiple attribute decision-making. *Reports in Mechanical Engineering*.

[18] Ali Z., Mahmood T., Mahmood K., Ullah Q., Khan Q. (2021). Einstein geometric aggregation operators using a novel complex interval-valued pythagorean fuzzy setting with application in green supplier chain management. *Reports in Mechanical Engineering*.

[19] Sahu R., Dash S., Das S. (2021). Career selection of students using hybridized distance measure based on picture fuzzy set and rough set theory. *Decision Making: Applications in Management and Engineering*.

[20] Wang L., Garg H., Li N. (2021). Pythagorean fuzzy interactive Hamacher power aggregation operators for assessment of express service quality with entropy weight. *Soft Computing*.

[21] Ullah K., Mahmood T., Garg H. (2020). Evaluation of the performance of search and rescue robots using T-spherical fuzzy hamacher aggregation operators. *International Journal of Fuzzy Systems*.

[22] Hara T., Uchiyama M., Takahasi S.-E. (1998). A refinement of various mean inequalities. *Journal of Inequalities and Applications*.

[23] Qin J. (2017). Interval type-2 fuzzy Hamy mean operators and their application in multiple criteria decision making. *Granular Computing*.

[24] Wu L., Wang J., Gao H. (2019). Models for competiveness evaluation of tourist destination with some interval-valued intuitionistic fuzzy Hamy mean operators. *Journal of Intelligent and Fuzzy Systems*.

[25] Wu L., Wei G., Gao H., Wei Y. (2018). Some interval-valued intuitionistic fuzzy Dombi Hamy mean operators and their application for evaluating the elderly tourism service quality in tourism destination. *Mathematics*.

[26] WeiZhang C., Zhang Y. (2019). Some q ‐rung orthopair fuzzy Hamy mean operators in multiple attribute decision‐making and their application to enterprise resource planning systems selection. *International Journal of Intelligent Systems*.

[27] Liu A., Božanić D., Milić A., Tešić D., Salabun W., Pamučar D. (2021). D numbers - fucom - fuzzy rafsi model for selecting the group of construction machines for enabling mobility. *Facta Universitatis – Series: Mechanical Engineering*.

[28] Wang Y. (2019). Intuitionistic fuzzy interaction Hamy mean operators and their application to multi-attribute group decision making. *Group Decision and Negotiation*.

[29] Sinani F., Erceg Z., Vasiljević M. (2020). An evaluation of a third-party logistics provider: the application of the rough Dombi-Hamy mean operator. *Decision Making: Applications in Management and Engineering*.

[30] Liu X. (2019). Linguistic intuitionistic fuzzy hamy mean operators and their application to multiple-attribute group decision making. *IEEE Access*.

[32] Zhang Y. (2020). Models for multiple attribute decision making with fuzzy number intuitionistic fuzzy hamy mean operators and their application. *IEEE Access*.

[33] Liu P., Xu H., Geng Y. (2020). Normal wiggly hesitant fuzzy linguistic power Hamy mean aggregation operators and their application to multi-attribute decision-making. *Computers & Industrial Engineering*.

[34] Wei G., Wang J., Wei C., Wei Y., Zhang Y. (2019). Dual hesitant pythagorean fuzzy hamy mean operators in multiple attribute decision making. *IEEE Access*.

[35] Li Z., Gao H., Wei G. (2018). Methods for multiple attribute group decision making based on intuitionistic fuzzy Dombi Hamy mean operators. *Symmetry*.

[36] Karamaşa Ç., Karabasevic D., Stanujkic D., KookhdanKookhdan A. R., MishraMishra A. R., Ertürk M. (2021). An extended single-valued neutrosophic AHP and MULTIMOORA method to evaluate the optimal training aircraft for flight training organizations. *Facta Universitatis – Series: Mechanical Engineering*.

[37] Li Z., Wei G., Lu M. (2018). Pythagorean fuzzy hamy mean operators in multiple attribute group decision making and their application to supplier selection. *Symmetry*.

[38] Sindhu M. S., Rashid T., Kashif A. (2021). Multiple criteria decision making based on Hamy mean operators under the environment of spherical fuzzy sets. *Journal of Intelligent and Fuzzy Systems*.

[39] Rani D. (2020). Robust averaging-geometric aggregation operators for complex intuitionistic fuzzy sets and their applications to MCDM process. *Arabian Journal for Science and Engineering*.

[40] Rani H., Rani D. (2020). Novel aggregation operators and ranking method for complex intuitionistic fuzzy sets and their applications to decision-making process. *Artificial Intelligence Review*.

[41] Xu B. (2020). Methods for evaluating the computer network security with fuzzy number intuitionistic fuzzy dual Hamy mean operators. *Journal of Intelligent and Fuzzy Systems*.

[42] Mandal U. (2021). Intuitionistic fuzzy Dombi aggregation operators and their application to multiple attribute decision-making. *Granular Computing*.

[43] Ali R., Abdullah S., Muhammad S., Naeem M., Chinram R. (2021). Complex intuitionistic fuzzy Maclaurin symmetric mean operators and its application to emergency program selection. *Journal of Intelligent and Fuzzy Systems*.

[44] Fan C.-L., Song Y., Fu Q., Lei L., Wang X. (2018). New operators for aggregating intuitionistic fuzzy information with their application in decision making. *IEEE Access*.

[45] Ullah K. (2021). Picture fuzzy maclaurin symmetric mean operators and their applications in solving multiattribute decision-making problems. *Mathematical Problems in Engineering*.

[46] Hussain A., Alsanad A., Ullah K., Ali Z., Jamil M. K., Mosleh M. A. A. (2021). Investigating the short-circuit problem using the planarity index of complex q-rung orthopair fuzzy planar graphs. *Complexity*.

[47] Mahmood T. (2020). A novel approach towards bipolar soft sets and their applications. *Journal of Mathematics*.

[48] Mahmood T., Ullah K., Khan Q., Jan N. (2019). An approach toward decision-making and medical diagnosis problems using the concept of spherical fuzzy sets. *Neural Computing & Applications*.

[49] Riaz M., Çagman N., Çagman N., Wali N., Mushtaq A. (2020). Certain properties of soft multi-set topology with applications in multi-criteria decision making. *Decision Making: Applications in Management and Engineering*.

[50] Ali Z., Mahmood T., Yang M.-S. (2020). Complex T-spherical fuzzy aggregation operators with application to multi-attribute decision making. *Symmetry*.

[51] Tamir D. E., Rishe N. D., Kandel A. (2015). Complex fuzzy sets and complex fuzzy logic an overview of theory and applications. *Fifty Years of Fuzzy Logic and its Applications*.

[52] Bi L., Dai S., Hu B., Li S. (2019). Complex fuzzy arithmetic aggregation operators. *Journal of Intelligent and Fuzzy Systems*.

[53] Rani D. (2019). Complex interval-valued intuitionistic fuzzy sets and their aggregation operators. *Fundamenta Informaticae*.

